# *Plasmodium falciparum* Rosetting Epitopes Converge in the SD3-Loop of PfEMP1-DBL1α

**DOI:** 10.1371/journal.pone.0050758

**Published:** 2012-12-05

**Authors:** Davide Angeletti, Letusa Albrecht, Karin Blomqvist, María del Pilar Quintana, Tahmina Akhter, Susanna M. Bächle, Alan Sawyer, Tatyana Sandalova, Adnane Achour, Mats Wahlgren, Kirsten Moll

**Affiliations:** 1 Department of Microbiology, Tumor- and Cellbiology (MTC), Karolinska Institutet, Stockholm, Sweden; 2 Escuela de Medicina y Ciencias de la Salud, Facultad de Ciencias Naturales y Matemáticas, Universidad del Rosario, Bogotá, Colombia; 3 Center for Infectious Medicine, Department of Medicine, Karolinska University Hospital Huddinge, Karolinska Institutet, Stockholm, Sweden; 4 EMBL Monoclonal Antibodies Core Facility, Monterotondo-Scalo (RM), Italy; Universidade Federal de Minas Gerais, Brazil

## Abstract

The ability of *Plasmodium falciparum* parasitized RBC (pRBC) to form rosettes with normal RBC is linked to the virulence of the parasite and RBC polymorphisms that weaken rosetting confer protection against severe malaria. The adhesin PfEMP1 mediates the binding and specific antibodies prevent sequestration in the micro-vasculature, as seen in animal models. Here we demonstrate that epitopes targeted by rosette disrupting antibodies converge in the loop of subdomain 3 (SD3) which connects the h6 and h7 α-helices of PfEMP1-DBL1α. Both monoclonal antibodies and polyclonal IgG, that bound to epitopes in the SD3-loop, stained the surface of pRBC, disrupted rosettes and blocked direct binding of recombinant NTS-DBL1α to RBC. Depletion of polyclonal IgG raised to NTS-DBL1α on a SD3 loop-peptide removed the anti-rosetting activity. Immunizations with recombinant subdomain 1 (SD1), subdomain 2 (SD2) or SD3 all generated antibodies reacting with the pRBC-surface but only the sera of animals immunized with SD3 disrupted rosettes. SD3-sequences were found to segregate phylogenetically into two groups (A/B). Group A included rosetting sequences that were associated with two cysteine-residues present in the SD2-domain while group B included those with three or more cysteines. Our results suggest that the SD3 loop of PfEMP1-DBL1α is an important target of anti-rosetting activity, clarifying the molecular basis of the development of variant-specific rosette disrupting antibodies.

## Introduction

The ability of *P. falciparum* parasitized RBC (pRBC) to form rosettes, clustering of uninfected erythrocytes around pRBC, is an adhesion property that varies in-between isolates which has also been found associated with the virulence of the parasite [1], [2], [3], [4], [5], [6], [7]. Rosetting is linked to cerebral malaria [2], [7] and other forms of severe disease [6], [8], [9], [10], and experimental models suggest that rosetting enhances microvascular obstruction [11], [12], thought to be a key pathological process in severe malaria. The importance of rosetting is underlined by the fact that host erythrocyte polymorphisms that weaken rosetting (blood group O, low levels of CR1, HbS) also confer protection against severe malaria [1], [2], [3], [5], [13], [14]. Blood group O is associated with a 66% reduction in the odds of developing severe malaria compared with the non-O blood groups [6], [8], [9], [10]. The vaso-occlusive effects of rosetting have consequently been suggested to be important for the development of severe malaria [15].

Rosetting is mediated by the interaction of the parasite ligand *Plasmodium falciparum* erythrocyte membrane protein 1 (PfEMP1) with serum-proteins and receptors on the human RBC surface [6], [7]. PfEMP1 polypeptides share a common structure with an N-Terminal Sequence (NTS) followed by tandemly arranged Duffy Binding Like domains (DBL) and Cysteine-rich InterDomain Regions (CIDR) [16], [17], [18]. The N-terminal NTS-DBL1α-domain is central for binding to host RBC [3], [19], [20], [21] and antibodies to this domain both disrupt rosettes *in vitro* and protect against the sequestration of pRBC *in vivo* targeting PfEMP1 [22], [23], [24], [25]. Previous work has also suggested NTS-DBL1α to be involved in the binding to the erythrocyte- and endothelial receptor heparan sulfate and the related glycosaminoglycan heparin has been found to disrupt rosettes *in vitro* and dislodge already sequestered pRBC into circulation *in vivo* [12], [20], [21]. PfEMP1-variants linked to rosetting are consequently important vaccine candidates.

Recent studies have shown that rosette disrupting antibodies are variant specific [26], but cross-reactivity can be observed in-between parasites of the same rosetting-variant with antibodies raised against whole NTS-DBL1α domains [27]. To identify and characterize structures in the NTS-DBL1α-domain of PfEMP1 critical for rosetting we have here generated sets of recombinant NTS-DBL1α-proteins that mediate rosetting and antibodies that disrupt rosettes. The reactivity of rosette-inhibitory mAbs was found to be predominantly localized in the loop of subdomain 3 (SD3) which connects the h6 and h7 α-helices of NTS-DBL1α, establishing this part as a key-region of PfEMP1 for induction of anti-rosetting antibodies and link it to protection against severe malaria. Analysis of polyclonal IgGs and human sera supported the importance of antibodies targeting these epitopes also during the acquisition of protective immunity. Our results therefore suggest the SD3-loop of NTS-DBL1α to be an immunodominant site of the PfEMP1 molecule important for protection against rosetting.

## Results

### Antibodies to the NTS-DBL1α-domain Recognize Epitopes on Live pRBCs

**Figure 1:**
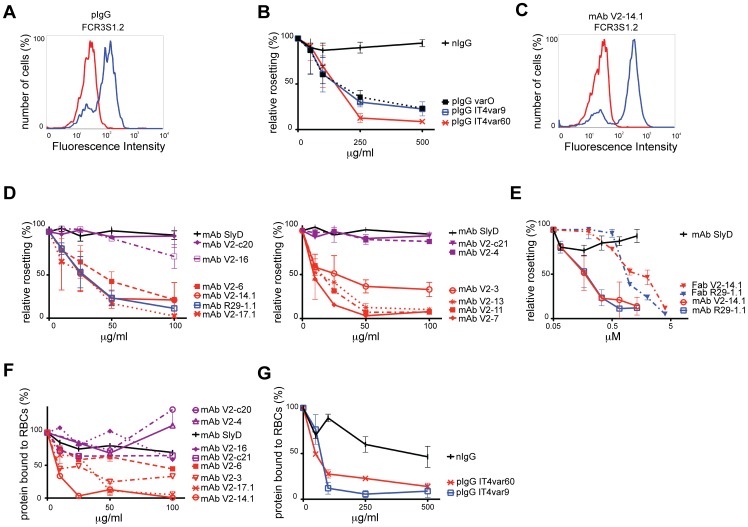
Characterization of antibodies towards the NTS-DBL1α-domain of rosette associated PfEMP1 molecules. A : Example of surface reactivity of a pIgG (at 10 µg/ml) with FCR3S1.2 pRBC as detected by Alexa488-conjugated secondary antibody and visualized by flow cytometry. pIgG and control nIgG are in blue and red respectively. **B:** Capacity of pIgG and sera to disrupt rosettes of the homologous parasite strain. Antibodies were tested at different concentrations from 10 to 500 µg/ml. Presented are the rosetting levels relative to a control incubated with 100 µg/ml nIgG. Three different experiments were performed in duplicate and bars indicate ± SD. **C**: Example of surface reactivity of a mAbs (at 20 µg/ml) towards the SD3-loop with FCR3S1.2 pRBCs as detected by Alexa488-conjugated secondary antibody and visualized by flow cytometry. mAbV2–14.1 and control mAbSlyD are in blue and red respectively. **D**: Capacity of the mAbs to disrupt rosettes of the homologous parasite strain. Antibodies were tested at different concentrations from 5 to 100 µg/ml. Presented are rosetting levels relative to a control incubated with 100 µg/ml mAb SlyD. Three different experiments were performed in duplicate and bars indicate ± SD. **E**: Capacity of Fab fragments and the corresponding mAbs to disrupt rosettes of the homologous parasite strain. Antibodies were tested at different concentrations from 0.06 to 4 µM. Presented are rosetting levels relative to a control incubated with 1 µM mAb SlyD. Three different experiments were performed in duplicate and bars indicate ± SD. **F:** Inhibition of NTS-DBL1α-domain binding to RBC by mAbs. Recombinant NTS-DBL1α was incubated with different concentrations of mAbs and thereafter assayed for its capacity to bind to human RBC. Binding was detected with anti-his mAb followed by Alexa488-conjugated secondary antibody by flow cytometry. Binding is expressed as relative binding as compared to the control mAb SlyD. Three different experiments were performed and bars indicate ± SD. **G:** Inhibition of NTS-DBL1α-domain binding to RBC by homologous pIgG. Assays were carried out as described under F. Three different experiments were performed and bars indicate ± SD.

To identify and characterize the structures in PfEMP1 that mediate rosetting we expressed three different species of NTS-DBL1α of the rosetting parasites FCR3S1.2 (expressing the PfEMP1 encoded by the IT4var60 gene), R29 (expressing the PfEMP1 encoded by the IT4var9 gene) and PaloAlto varO as histidine-tagged proteins in *E.coli*. The solubilized proteins were purified to homogeneity and used for the production of sera in goats, from which the polyclonal IgGs (pIgG) were purified. The pIgGs reacted in ELISA with the corresponding recombinant protein and labeled the surface of live RBC, infected with the homologous parasites, in flow cytometry while IgG from non-immunized goats (nIgG) did not (Fig. 1A and Fig. S2A). The reactive antibodies specifically labeled the pRBC-surface and disrupted the rosettes of the homologous parasite in a concentration dependent manner (Fig. 1B).

Three groups of BALB/c-mice were subsequently immunized with recombinant NTS-DBL1α to produce mAbs; two groups with the recombinant protein from IT4var60 and one group with the protein from IT4var9 (Table 1). The reactivity of the mAbs, which were positive with the fusion-proteins in ELISA, was assessed with the live pRBC surface in flow cytometry and in rosette disruption assays (Fig. 1C and D and Fig. S2B). A total of nine surface reactive mAbs were generated after immunizations with NTS-DBL1α of IT4var60 while immunization with NTS-DBL1α of IT4var9 resulted in one surface reactive mAb. Two different functional groups could be distinguished among the surface reactive antibodies: eight out of ten (80%) were found to dose-dependently disrupt rosettes while two of them did not have anti-rosetting activity (mAbV2–4 and mAb V2–16) (Fig. 1D). The Fab-fragments of the corresponding mAbs were also found to disrupt rosettes while a control mAb did not (Fig. 1E).

**Table 1:** Characterization of the antibodies used in the study.

Monoclonal antibodies							
Antibody	Immunogen(NTS-DBL1α)	Isotype of antibody	Rosette disruption(homologous pRBC)	Surface Reactivity (homologous pRBC)	ELISA-reactivity(homologous recombinant NTS-DBL1α domains)		Reactivity in peptide array
mAbV2–3	IT4var60/FCR3S1.2var2	IgG2b, κ	+	+	+		SD3-loop
mAbV2–6	IT4var60/FCR3S1.2var2	IgG2b, κ	+	+	+		SD3-loop
mAbV2–11	IT4var60/FCR3S1.2var2	IgG2a, κ	+	+	+		SD3-loop
mAbV2–14.1	IT4var60/FCR3S1.2var2	IgG2b, κ	+	+	+		SD3-loop
mAbV2–13	IT4var60/FCR3S1.2var2	IgG2a, κ	+	+	+		SD3-loop
mAbR29–1.1	IT4var9/R29var1	IgG2b, κ	+	+	+		SD3-loop
mAbV2–7	IT4var60/FCR3S1.2var2	IgG2a, κ	+	+	+		SD3-loop
mAbV2–17.1	IT4var60/FCR3S1.2var2	IgG2a, κ	+	+	+		–
mAbV2–4	IT4var60/FCR3S1.2var2	IgG2a, κ	–	+	+		–
mAbV2–16	IT4var60/FCR3S1.2var2	IgG2a, κ	–	+	+		–
mAbV2-c21	IT4var60/FCR3S1.2var2	IgG2a, κ	–	–	+		SD3-loop (YCSGDG)
mAbV2-c20	IT4var60/FCR3S1.2var2	IgG2b, κ	–	–	+^1^		SD3-H7
mAbR29-c3	IT4var9/R29var1	IgG2a, κ	–	–	+^1^		SD3-H7
mAbR29-c4	IT4var9/R29var1	IgM, κ	–	–	+^1^		SD2 (LARSFADIG)
**Polyclonal antibodies**							
Goat FCR3S1.2	NTS-DBL1α IT4var60	IgG	+	+	+	+	
Goat R29	NTS-DBL1α IT4var9	IgG	+	+	+	+	
Goat PAvarO	NTS-DBL1α PAvarO	IgG	+	+	+	+	
Rat SD1	SD1 of DBL1α IT4var60	serum	–	+	+	nd	
Rat SD2	SD2 of DBL1α IT4var60	serum	–	+	+	nd	
Rat SD3	SD3 of DBL1α IT4var60	serum	+	+	+	nd	

1 mAbV2-c20, mAbR29-c3 and mAbR29-c4 reacted in addition with the heterologous recombinant NTS-DBL1α domains of PAvarO and TM284S2.

### Antibodies Prevent the Binding of NTS-DBL1α to the RBC Surface

The proteins were assayed for binding to erythrocytes, in order to study how the antibodies influenced the interaction of the recombinant NTS-DBL1α-domain with RBC receptors. 10 µM of recombinant NTS-DBL1α was incubated with human O^+^ RBCs and the bound protein was visualized by flow cytometry. Concentration-dependent binding of the proteins was detected with the recombinant NTS-DBL1α of IT4var60 and NTS-DBL1α of IT4var9 (Fig. S1). While pre-incubation of the homologous recombinant protein with two of the mAbs specific for FCR3S1.2 (mAbV2–14.1, mAbV2–17.1) efficiently and dose-dependently prevented binding, the effects of mAbV2–6 and mAbV2–3 were less prominent (Fig. 1F) possibly due to differences in the fine specificities for the NTS-DBL1α (see below). There was no significant inhibition of binding with the non-rosette disruptive antibodies mAbV2–4, mAbV2–16, mAbV2-c20 or mAbV2-c21 (Fig. 1F) suggesting that the binding of the recombinant NTS-DBL1α to RBCs mimics parasite rosetting. The pIgGs also blocked binding of recombinant NTS-DBL1α domains of FCR3S1.2 and R29 to RBCs (Fig. 1G). No or minor effects on binding were seen with control antibodies.

### Rosetting Epitopes are Spatially Conserved

**Figure 2:**
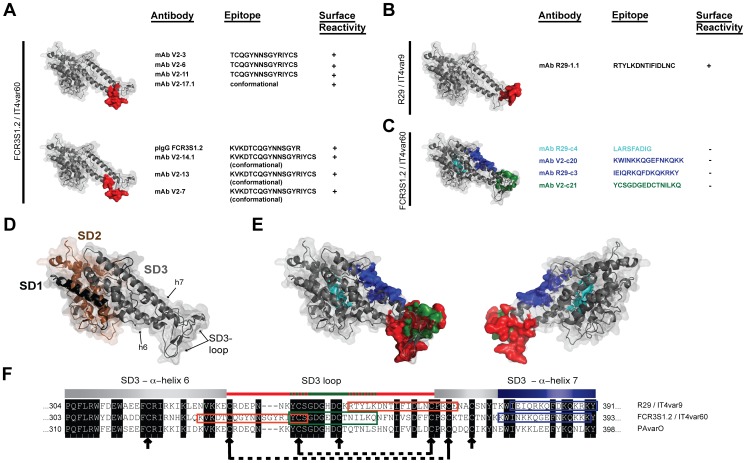
Identification a loop containing functionally important epitopes in subdomain 3 of the NTS-DBL1α-domain. A : Molecular model of the NTS-DBL1α-domain of IT4var60, based on NTS-DBL1α-PAvarO crystal structure [29], showing the localization of epitopes targeted by anti-rosetting antibodies (in red). **B:** Molecular model of the NTS-DBL1α-domain of IT4var9, based on NTS-DBL1α-PAvarO crystal structure [29], showing the localization of epitopes targeted by anti-rosetting antibodies (in red). **C**: Molecular model of the NTS-DBL1α-domain of IT4var60, based on NTS-DBL1α-PAvarO crystal structure [29], showing the localization of epitopes targeted by non surface reactive antibodies (in blue and green). **D:** Representation of the molecular model of NTS-DBL1α-domain of IT4var60 with the three subdomains (SD) 1, 2 and 3 in black, brown and grey, respectively. **E:** Two orthogonal views of the molecular model of NTS-DBL1α-domain of IT4var60 with surface exposed epitopes in red and non-surface exposed epitopes in blue and green. **F**: Alignment of the SD3 sequences of three rosette-associated NTS-DBL1α-domains. The conserved amino acid residues are in black. The recognition-sites of the mAbs mapped to this region are shown in color; epitopes in red are available on the surface of pRBCs while the ones in blue and green are hidden. Arrows and dashed lines indicate cysteines and disulfide bridges, respectively.

There is currently little data available as to what epitopes are targeted by anti-rosetting antibodies. We therefore developed a peptide array holding seven complete NTS-DBL1-sequences (Table S1). It covered 400–450 aa per NTS-DBL1 domain with ≈100 overlapping 15-mer peptides per sequence. Ten of the mAbs that showed surface reactivity and four surface-negative mAbs were studied for binding in the arrays. Four distinct sets of linear epitopes as well as two overlapping conformational epitopes were identified for the mAbs tested (Fig. 2). Three antibodies that showed surface- and rosette disruption activity with pRBC of FCR3S1.2 (mAbV2–3, mAbV2–6, mAbV2–11) bound to the 15-mer peptide TCQGYNNSGYRIYCS of IT4var60, a sequence localized within the loop that connects the two α-helices h6 and h7 of subdomain 3 (SD3) of DBL1α (Fig. 2A and D and Fig. S3A). This sequence is also part of Homology Block HB12, which is partially conserved among DBLα and DBLγ domains as recently described by Rask et al. [28].

Four biologically active mAbs (mAbV2–14.1, mAbV2–13, mAbV2–7 and mAbV2–17.1) could not be mapped for reactivity in the linear peptide array indicating that they possibly recognize conformational epitopes (Fig. S3A). However three of them (mAbV2–14.1, mAbV2–13 and mAbV2–7) were found to bind a 19aa loop-sequence (KVKDTCQGYNNSGYRIYCS) when loop-peptides longer than 15aa were studied for binding (Fig. 2A and Fig. S4C). One mAb (mAbV2–17.1) was found to react with epitopes related to the SD3-loop as demonstrated by antibody competition experiments with pRBCs of FCR3S1.2 (Fig. S4A and S4B). The data suggested that the epitopes recognized by mAbsV2–17.1 and mAbV2–14.1 were not identical to that of the inhibitory mAbs that bind to the linear SD3 epitopes, but spatially related.

A third group of mAbs (mAbV2–4 and mAbV2–16) displayed surface-reactivity but did not disrupt rosettes and could not be mapped in the linear peptide array (Fig. S3A and S4C) suggesting a different epitope-specificity as compared to the mAbs of the first group.

A fourth subset of mAbs (mAbV2-c20 and mAbV2-c21) did not react with the pRBC surface and did not confer any anti-rosetting activity (Fig. 1D and Fig. S2B). mAb-c20 reacted in the microarray with a stretch of residues localized on the α-helix h7 (KWINKKQGEFNKQKK; Fig. 2C and E and Fig. S3A). It also showed reactivity with the homologous recombinant protein as well as cross-reactivity with two other NTS-DBL1α domains in ELISA (Table 1). The epitope corresponds to the second part of the HB5, as defined by Rask et al. [28], and is predicted to be surface-exposed [29]: we therefore explored the possibility that it is shielded by serum-proteins [30], [31], [32]. However, no binding of mAbV2-c20 to the pRBC surface could be detected, independently if the cells were stripped of serum proteins or studied directly after *in vitro* culture in the presence of 10% human serum (Fig. S2C) indicating that this epitope appears to be hidden. The epitope of the second mAb in this group (mAbV2-c21), was mapped to 15aa present in SD3-loop (YCSGDGEDCTNILKQ; Fig. 2C and E and Fig. S3A). This stretch of amino-acids corresponds to HB7 [28] and is fairly conserved and predicted to be only partially surface exposed, which is confirmed by our data (Fig. 2F).

In a complementary set of experiments we studied mAbR29–1.1, which binds to the surface of pRBC of R29, and two R29-specific mAbs that did not (mAbR29-c3, mAbR29-c4). The mAbR29–1.1epitope was mapped to the peptide RTYLKDNTIFIDLNC (Fig. S3B) that is localized in the SD3-loop of IT4var9-DBL1α (Fig. 2B) indicating that the biologically active antibodies described here target different variable regions within the same loop structure. Similarly to mAbV2-c20, a surface negative mAb (mAbR29-c3) bound to an amino acid stretch within h7 that is not exposed at the pRBC surface (Fig. 2C). A third surface negative mAb (mAbR29-c4) bound to the non-exposed, conserved LARSFADIG motif present in subdomain 2 (SD2; Fig. 2C) and was in addition cross-reactive with two other NTS-DBL1α domains in ELISA.

### SD3 of PfEMP1-DBL1α is the Target of Rosette-inhibitory Antibodies

**Figure 3:**
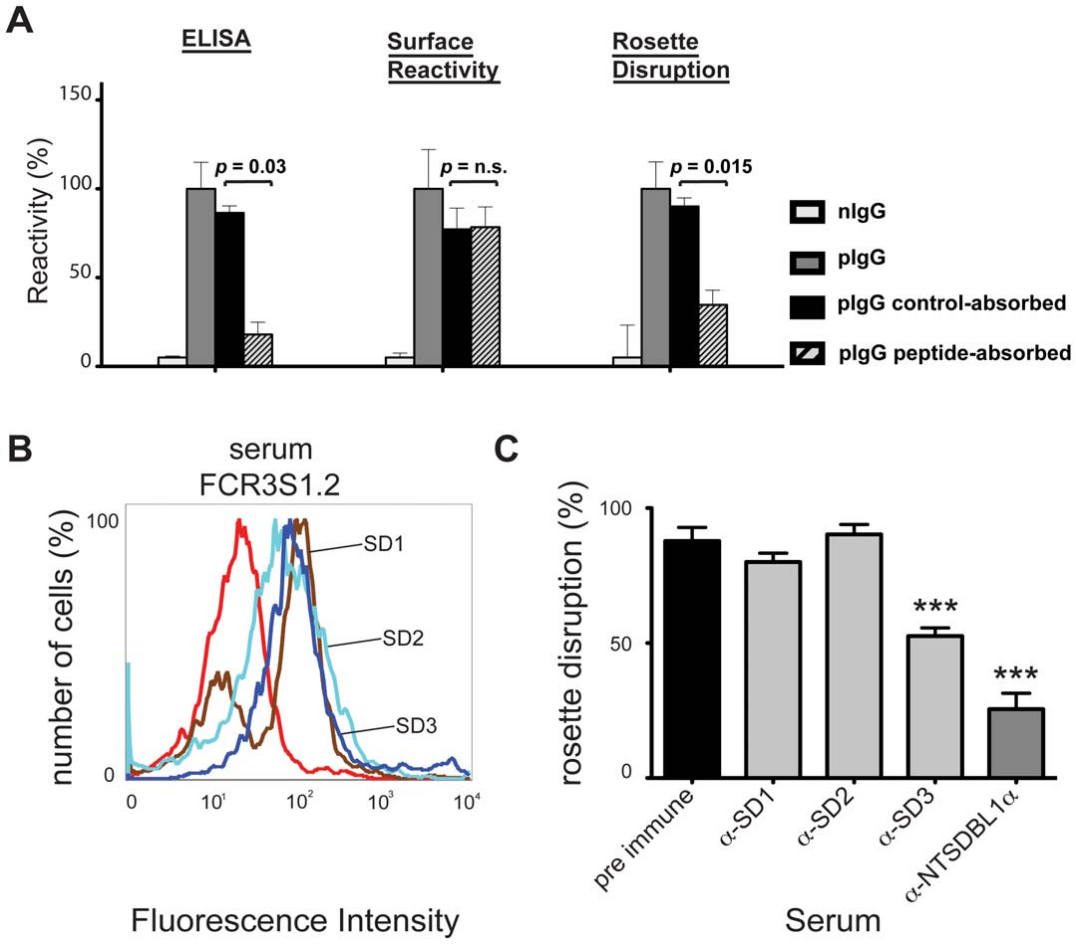
Importance of the SD3 of PfEMP1-DBL1α in anti-rosetting response. A: Residual activity of pIgG_IT4var60_ after absorption on the SD3-loop peptide of NTS-DBL1α of IT4var60 (KVKDTCQGYNNSGYRIYCS). ELISA plates were coated with 5 µg/ml of peptide and the absorption of the pIgGs was verified by adding 10 µg/ml of the different pIgGs followed by ALP-conjugated secondary antibody. The peptide-absorbed/non-absorbed pIgGs were tested for surface reactivity by flow cytometry at 10 µg/ml and for capacity to disrupt rosettes of the homologous parasite at 250 µg/ml. As control pIgG were absorbed on the same peptide with scrambled sequence (CTSSKDYIYVQGCNNRGYK). All results are shown as relative reactivity as compared to pIgG (set to 100%). Bars show the mean of six independent experiments ± SD. **B:** pRBC surface reactivity of sera from rats on FCR3S1.2/IT4var60 as detected by an Alexa488-conjugated secondary antibody and visualized by flow cytometry. The rats were immunized with subdomain 1 (SD1; aa 1–119; brown), subdomain 2 (SD2; aa 120–272; light blue) or subdomain 3 (SD3; aa 273–393; dark blue) of IT4var60. Reactivity of a pre-immune rat serum is shown in red. **C:** Rosette disruption activity of sera of rats immunized as described under A or with full length NTS DBL1α (dilution 1∶5). Presented are the rosetting levels relative to a control incubated with PBS. Six different experiments were performed in duplicate and bars indicate SEM (Standard error of the mean). *** = p<0.001 as compared to pre-immune serum.

The importance of the SD3-loop as a target for biologically active antibodies was established by the reactivity of the pIgG generated towards the NTS-DBL1α-domain of IT4var60 as seen in our peptide array. Among the 10 peptides showing the highest Ab reactivity, only one represented the SD3 loop while the others were derived from subdomain 1 (SD1; Fig. S3C). We therefore absorbed the pIgG over NHS-activated agarose beads coupled to the SD3-loop sequence and found that ≈70% of the anti-rosetting effect was removed with a concurrent decrease of the ELISA response towards the peptide, while the pRBC surface reactivity remained unaltered (Fig. 3A). Absorption of the same pIgG on a scrambled SD3-loop sequence (CTSSKDYIYVQGCNNRGYK) did not influence the rosette disruption activity and hence confirmed the specificity and excluded possible unspecific absorption due to charge effects (Fig. 3A).

In order to investigate whether the SD3 of IT4var60 (FCR3S1.2) by itself could generate biologically active antibodies, as does the complete NTS-DBL1α, we expressed the three distinct subdomains (SD1, SD2 and SD3) of IT4var60 (FCR3S1.2var2) as his-tagged fusion proteins and immunized rats. All the induced Abs bound to the pRBC-surface but only SD3-specific Abs disrupted rosettes to ≈50% (Fig. 3B and C) suggesting that the SD3-loop of DBL1α is involved in the induction of anti-rosetting responses.

### Recognition of SD3-loop Peptides by Human Sera

**Figure 4:**
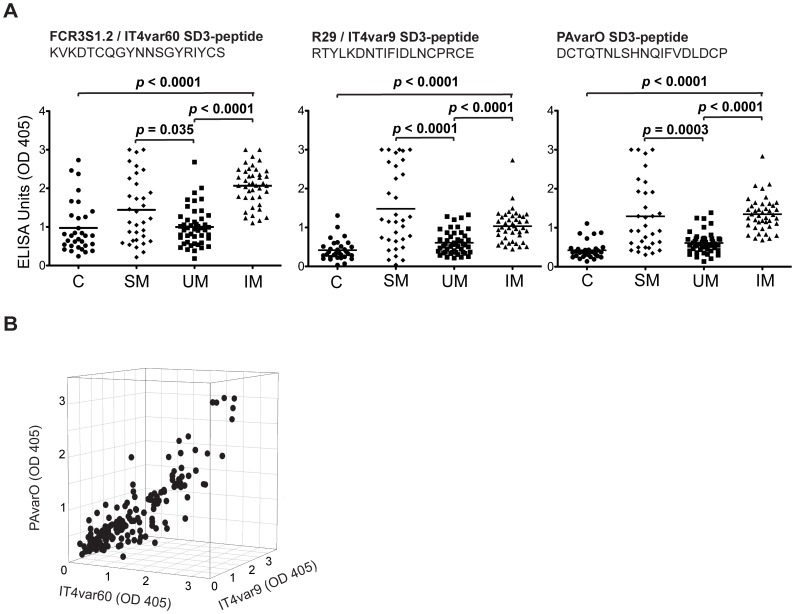
Individuals living in endemic areas acquire antibodies against SD3-loop sequences. A: IgGs levels in human sera (diluted 1∶1000) against the NTS-DBL1α SD3-loop peptides from parasites IT4var60, IT4var9 and PAvarO as detected by ELISA. IgG levels were measured in 32 non-immune Swedish controls(C), 33 patients with severe malaria (SM), 47 patients with uncomplicated malaria (UM) and 40 immune adults (IM). **B:** Correlation between patient-serum IgG reactivity as measured by ELISA with the SD3-loop peptides KVKDTCQGYNNSGYRIYCS (IT4var60), DCTQTNLSHNQIFVDLDCP (PAvarO) and RTYLKDNTIFIDLNCPRCE (IT4var9). The sera were tested for reactivity towards the three distinct peptides in ELISA (as described under B) and the correlation was tested using non-linear regression; R^2^ = 0.75, p<0.0001.

Human sera diluted 1∶1000 showed reactivity with the identified SD3-peptides. The levels of IgG were measured by ELISA in sera of 47 patients with uncomplicated malaria, 33 patients with severe malaria, 40 malaria immune adults and 32 non-immune Swedish controls (Fig. 4A). Anti-SD3-peptide antibody levels were high in some children with severe malaria but these sera did not disrupt rosettes while the IgG-levels were less pronounced in children with mild disease (Fig. 4A). Mean IgG antibody-levels were still the highest in sera of the immune adults for two of the SD3 peptides but not for that of IT4var9, (p<0.0001). We also established that the responses in the sera were co-correlated (R^2^ = 0.75, p<0.0001; Fig. 4B).

### Bioinformatics Analysis of NTS-DBL1α Sequences

**Figure 5:**
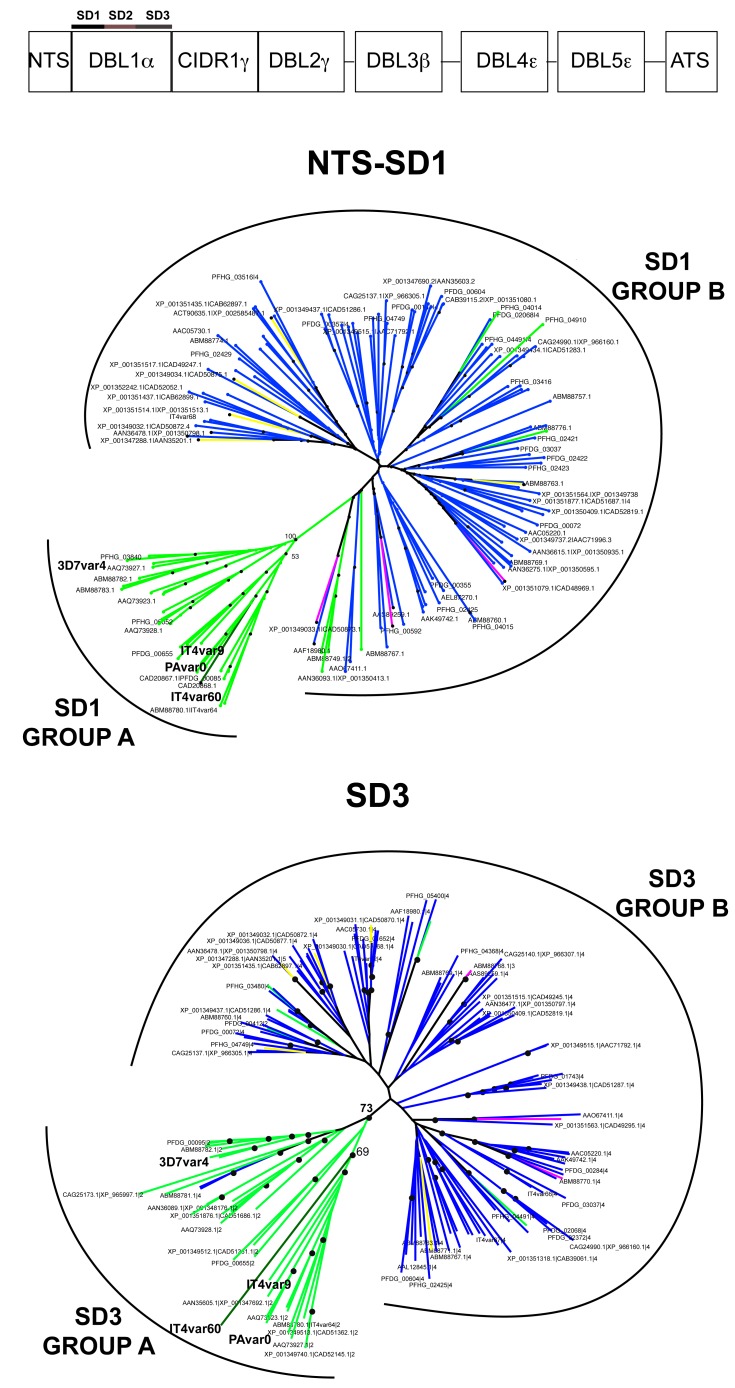
Phylogenetic tree of NTS-SD1 and SD3 sequences. The Neighbor Joining tree shows segregation of the NTS-SD1 (considered from h1 to LARSFADIG) and SD3 (considered from h6 to h7) in two groups. Bootstrap support, after 1000 replicates, is only shown for the branches separating different groups, black dots at nodes indicate bootstrap values above or equal to 50%. 1-cys sequences are colored in dark green, 2-cys in green, 3-cys in pink, 4-cys in blue and 5-cys in yellow.

Neighbor Joining distance trees were built based on amino acid alignment of 144 SD1, SD2 or SD3 sequences (from NTS-h1 including the conserved LARSFADIG sequence, h2 to h5 or h6 to h7 of NTS-DBL1α, respectively). The majority of PfEMP1-DBL1α sequences from patients isolates available today lack the sequence of the SD3 region. Therefore, only sequences of laboratory parasites strains 3D7, IT4, Dd2 and HB3 were used for the analysis. The sequences could be divided in two major groups (Fig. 5 and Fig. S5A). This phylogenetic grouping was found to be associated with the number of cysteine residues present in SD2 of DBL1α, and resembles the previos grouping obtained according to the SD2 sequence [33]. Of the 144 SD2-sequences one had one cysteine residue, 34 sequences had 2, three had 3, 101 had 4 and five had 5. The ≤2 cys (DBLα1) sequences fell into one group (SD1 and SD3 group A) while sequences with ≥3 cys fell into the second group (SD1 and SD3 group B). Sequence signatures of the different groups are shown in Figure S5B. Accordingly, ≈75% of sequences group into Group B, while ≈25% of sequences into Group A. Sequences of the rosetting parasites IT4var60, IT4var9 and PAvarO studied herein, as well as other sequences previously associated with severe parasite phenotypes, all fall into SD1-SD3-group A.

## Discussion

This study of erythrocyte-adhesive PfEMP1 polypeptides and rosette-inhibitory antibodies employs multiple chemical- and biological assays and presents two key findings: firstly, the importance of the loop of SD3 of the NTS-DBL1α-domain, a target of anti-rosetting activity and, secondly, the immunogenicity of the SD3-loop in the induction of antibodies (Fig. S6). These findings underline the importance of the SD3 domain in molecular events linked to the rosetting phenomena.

We report the generation of a number of antibodies to distinct NTS-DBL1α-domains that specifically bind to the surface of pRBCs that disrupt rosettes or antibodies that only stain the pRBC-surface. The specificities and the potencies of the mAbs were confirmed by the fact that also Fab fragments specifically reacted with the surface of the pRBCs and disrupted rosettes. The epitopes recognized by the mAbs that were able to disrupt rosettes were found to be localized in subdomain 3 and mainly within the loop, despite displaying some heterogeneity in their fine specificities. Conformational epitopes recognized by additional functional antibodies were also localized closely to the SD3-loop since their reactivities could be competed out with mAbs that bound to linear epitopes within the loop. Furthermore we were able to reduce the anti-rosetting activity of a pIgG directed to the whole NTS-DBL1α domain by 70% by depleting the specific antibodies on a SD3-loop peptide. Still, it is likely that several parts of the NTS-DBL1α head-structure are involved in erythrocyte binding since SD1 has previously been suggested to be involved in rosetting-binding [29], and since our sera from the animals immunized with SD3 could only partially disrupt rosettes. Recent reports argue that SD3 of PfEMP1 VAR2CSA binds to CSA [34] but important epitopes in VAR2CSA have also been found present outside of the SD3-loop [35], [36], [37]. Whatever the case, the data presented here show that the loop located in SD3 of the DBL1α-domain is a highly immunogenic part of PfEMP1 that is easily targeted by functional Ab independently of the parasite strain and the animal species used. In addition, the importance of the SD3 has recently been pointed out in the *P. vivax* DBP (PvDBPII) DBL domain where the SD3 is essential for PvDBPII binding to its receptor and mAbs targeting residues in SD3 are able to inhibit this interaction [38].

Most of our mAbs reacted both with the pRBCs surface and disrupted rosettes but two of them only bound to the pRBC surface suggesting that mere binding of an antibody to PfEMP1 does not disrupt rosetting. It is also unlikely that our antibodies sterically hinder the binding to the rosetting receptor at a distance since the Fab fragments effectively inhibited rosetting. Taken together with the pIgG peptide-absorption data and results from immunizations with the three distinct subdomains (Fig. 3), this argues that rosette-disruption is not due to distance-induced conformational changes in PfEMP1 upon Ab binding but rather receptor inhibition.

As reported for other pathogens and proteins [39], [40], [41] antibodies commonly target immunodominant variable regions located in proximity of conserved ligand binding-sites thus inhibiting the interaction. Notably in the proximity of the SD3-loop in h6, h7 and in the loop itself there are several basic residues (arginine, histidine, lysine) that could be key for the interaction of the NTS-DBL1α domain with the negatively charged erythrocyte receptor heparan sulfate [20], [29], [42]. HB7 and HB12, which are part of the SD3 loop, have also been suggested to carry motifs specific for receptors adhesion [28]. Other studies also suggest that receptor-binding sites are present in SD3 for DBL1α in the binding to CR1 [43]. Yet different binding sites were identified for the DBL domain of EBA-175-binding to Glycophorin A [44] and for DBP-binding of *P. vivax* to DARC [45], [46] but in the latter case the SD3-loop was located in proximity of the RBC membrane upon receptor binding suggesting a possible role in adhesion as well.

The IgG levels of human sera to SD3-loop-peptides of individuals living in areas where *P. falciparum* is endemic were elevated, as compared to non-exposed adults. We found reactivity with the three SD3-loop-peptides independently of the geographical origin of the sera for all the groups of patients investigated. Unexpectedly, we measured higher antibody titers in patients with severe disease as compared to patients with uncomplicated malaria but the sera of the former group failed to disrupt rosettes showing that the antibodies measured by ELISA are not always functional. Although it is likely that only a fraction of the IgG measured was protective, it is tempting to speculate that human immunity is acquired in a stepwise manner where each individual infection with rosetting-parasites boosts the antibody response towards SD3-loop epitopes, protecting older children and immune adults against severe malaria.

In a previous study, three rosetting parasite strains were found to display variant specific surface epitopes and were grouped as multiple serotypes [26]. Our data confirm this finding and suggests that at least part of the molecular background to the serotypes is found in variable sequences in the conserved location within the SD3 of the DBL1α-domain. However, most sequence information that is available to date covers only 80–150 aa tags produced with primers located in the relatively conserved SD2 areas N-terminally to the SD3-loop [47], [48], [49]. This part of the molecule is indeed interesting since the number of cysteine residues present therein has been found associated with the rosetting phenotype of the parasite and the severity from malaria [48], [49]. The bioinformatic analysis presented here shows a similar clustering of the analyzed sequences whether we consider just the DBL1α-tag (between PoLV1 and PoLV4) of SD2 [48] or the SD3 part of DBL1α (from α-helix h6 to α-helix h7) (Fig. 5) and suggests that determinants in the SD3 sequence are also important and associated with disease severity and rosetting. The analysis is corroborated by a previous study by Rask et al. [28] where it is suggested that SD3 sequences belonging to the DBLα1 class are well supported as a subgroup, further subdividing it into two groups depending whether it is followed by CIDRα or CIDRγ. Further the authors demonstrated the presence of a recombination hotspot between SD2 and SD3, upstream of SD3-loop (HB7), hypothesizing independent function of SD1+SD2 and SD3.

Development of variant specific anti-rosetting antibodies appears to be the most common event in natural acquisition of immunity to parasites [26], however cross-reactivity of anti-sera to NTS-DBL1α in-between pRBC-surfaces of parasites was recently reported by Ghumra et al [27]. The latter has been shown to be extremely difficult to obtain in a laboratory and it is mainly induced by immunizations with NTS-DBL1α from IgM-binding parasites. Another possible rational design of a vaccine against severe malaria could include minimal regions with biological function. Our study now suggests a chimeric vaccine that could include a number of SD3-loops derived from rosetting parasites targeting different serological variants.

The data presented here support the notion that PfEMP1 sequences important for the acquisition of rosette-inhibitory antibodies are localized within the SD3-loop of the DBL1α domain. The efficacy of the Abs, the human serum-reactivity and the ease with which we could induce biologically active Abs to SD3 suggest this subdomain to be one of the major target for protective antibodies that prevent the phenomenon of rosetting.

## Materials and Methods

### Ethics Statement

For the collection of human samples written informed consent was obtained and the study was approved by Karolinska Institutets Regional Ethical Review Board (permission 03/095) and the Uganda National Council for Science and Technology (permission MV 717). Animal immunizations were carried out commercially by EMBL (Monterotondo, Italy) according to European Guidelines 86/609/EEC. Animal immunizations at Agrisera (Vännas, Sweden) were approved by the animal ethical committee at Umeå University (permissions A37–10 and A73–11) and followed the Swedish Regulations (1988∶534).

### Parasite Cultures

P. falciparum laboratory strains were cultivated according to standard methods [50], with modifications for patient isolates [49], [51]. The rosetting phenotype of FCR3S1.2 was maintained by enrichment over a Ficoll-gradient [50], while enrichment on mAbs was carried out as described for R29 and PAvarO [21].

### Production of Recombinant Protein

NTS-DBL1 domains were PCR-amplified and cloned into the pQE vector system (Qiagen) using specific primers (Table S2 for all vectors and primers used in the study). While genomic DNA was used for IT4var9 (R29var1) and SD3-IT4var60, *E. coli* codon-optimized DNA was applied for PAvarO [21] and IT4var60 (FCR3S1.2_var2_) [52]. NTS-DBL1α-domains were expressed as C-terminal 6x histidine-tagged recombinant proteins in the *E. coli* strain BL21 DE3 (ΔslyD). Production of protein was induced at OD_600_ = 0.6 with 0.1 mM IPTG. After 3 hours, the cells were lysed by sonication. Inclusion bodies (IB) were sampled using high speed centrifugation. The content of IB was solubilized in 6 M Guanidine HCl, 50 mM Tris-HCl pH 8, 100 mM NaCl, 10 mM EDTA pH 8, 10 mM DTT. All proteins were refolded by rapid dilution using ice-cold 200 mM Tris-HCl pH 8, 10 mM EDTA pH 8, 0.6 M L-arginine, 6.5 mM cysteamine, 3.7 mM cystamine. Refolded NTS-DBL1α-domains were thereafter dialyzed and concentrated using Amicon Ultracell centrifugal filter units (Millipore). All proteins were purified by Immobilized Metal Affinity Chromatography over Ni-NTA columns (Qiagen), eluted with 500 mM imidazole and further purified to homogeneity using size exclusion chromatography on a HiLoad 16/60 Superdex 75 pg column (GE-Healthcare). Correct folding was assessed by circular dichroism.

### Immunization of Animals and Generation of Monoclonal and Polyclonal Antibodies

Mouse monoclonal antibodies were produced at EMBL Monoclonal Antibody Core Facility, Monterotondo, Italy (http://www.embl.it/services/macf/index.html) as previously described [53]. Briefly, mice were immunized trice with 50 µg of recombinant protein, at one month interval. Serum levels of antibodies were measured in ELISA prior to fusion and a post fusion confirmatory ELISA was carried out for selection of positive cell clones. Monoclonality of the antibodies were either tested directly from the obtained cell supernatants of antibody producing cell clones or from purified from cell supernatants. After adjusting the pH to 7.4, purification was performed over a ProteinG agarose column (Pierce ThermoScientific, product#20399). Purified antibodies were used in the experiments after being dialyzed against PBS and subsequently concentrated. mAb SlyD, a mAb directed against an *E. coli* heat-shock protein, was produced in the same way and used as a control throughout the experiments.

Polyclonal antibodies were produced in goats and rats by Agrisera, Umeå, Sweden. Briefly, animals were immunized intramuscularly with 200 µg of protein four times at one month intervals; for the first immunization the protein was emulsified in Freund's complete while incomplete Freunds adjuvant was used for the remaining three; final bleed was carried out two weeks after the last immunization. From each goat approximately one liter of serum was obtained and the IgGs were purified over ProteinG columns.

### ELISA Assay

To assess the reactivity of each mAb with recombinant protein, ELISA assays were carried out as described [19]; plates were coated with 50 µl of 2.5 µg/ml protein overnight in carbonate buffer pH 9.6, subsequently blocked and thereafter incubated with 100 µl antibody containing solution in two different dilutions (mAbs cell supernatants 1∶20 and 1∶40; purified mAbs at 5 and 2.5 µg/ml). Reactivity was visualized using an anti-mouse IgG-ALP (dilution 1∶1000; Sigma-Aldrich) conjugated antibody; mAb SlyD was used as a control. For peptide ELISA the same protocol was used with the following modifications: 5 µg/ml of peptide was used for coating of plates, human serum was used at 1∶1000 dilution followed by anti-human IgG-ALP conjugated antibody (dilution1∶1000, Sigma-Aldrich).

### Analysis of Surface Reactivity of pRBC by Flow Cytometry

Trophozoite infected RBCs of ≈24–30 h p.i. were incubated with the pIgGs (final concentration 10 µg/ml), mAbs (final concentration 20 µg/ml) or rat sera (final dilution 1∶10) for 30 min in a volume of 50 µl. Non-immune goat IgG, mAb SlyD or pre-immune sera were used as controls in all experiments at the same concentrations. After incubation with the primary antibody, three washes with 200 µl PBS/2% FCS were performed followed by 30 min incubation with species-specific anti-IgG antibody coupled to ALEXA488 (dilution 1∶100, Invitrogen) in volumes of 50 µl. All antibodies were diluted in PBS/2% FCS. For nuclear staining, ethidium bromide was added at final concentration 2.5 µg/ml and pRBC were finally washed three times with 200 µl and resuspended in 200 µl PBS with 2% FCS. The cell acquisition was done using flow cytometry (FACSCalibur, BD Bioscience, http://www.bd.com) where 5000 pRBC were counted. The analysis was performed using FlowJo software. pRBCs incubated with the fluorophore-labelled secondary antibody only were used to define the non-reactive cell population; the percentage of positive cells was thereafter determined in all samples.

### Rosette Disruption Assay

To test the capacity of the pIgGs and mAbs to disrupt rosettes of the different parasite clones/strains 45 µl aliquots of well synchronized parasite culture 28–36 h p.i. were transferred to a 96 well plate and carefully mixed (using a pipette tip with wide opening, in order to avoid mechanical disruption of rosettes) with 5 µl of antibody solution. For pIgGs concentrations of 50, 100, 250 and 500 µg/ml, for mAbs concentrations of 10, 25, 50 and 100 µg/ml and for rat sera final dilution of 1∶5, 1∶10, 1∶20 and 1∶40 were applied; nIgG, mAb SlyD or pre-immune rat sera was analyzed at the same concentrations as control. Incubation was carried out for 60 min at RT, parasites were afterwards stained with Acridine Orange and the rosetting rate was counted in a fluorescent microscope. PRBC filling the RBC 1/3 or more were considered as potentially rosetting and included in the counting; counting of rosettes was carried out covering different areas of the slide to cover for unevenly spread rosettes [50].

### Erythrocyte Binding Assay

In order to analyze the capacity of the recombinant NTS-DBL1α-domains to bind RBCs the proteins were incubated for 30 min at RT with RBCs (O+) from Swedish donors in 50 µl PBS. After three washes with 200 µl PBS/2% FCS the complex was incubated with 50 µl mAb anti-his antibody (dilution 1∶200, Qiagen) for 30 min at RT. This step was followed by three washes with 200 µl PBS with 2% FCS and 30 min incubation with 50 µl goat-anti-mouse IgG antibody ALEXA488-conjugated (dilution 1∶100, Invitrogen). The complex was washed three times with 200 µl and re-suspended in 200 µl PBS with 2% FCS. The cell acquisition was done using flow cytometry (FACSCalibur, BD Bioscience, http://www.bd.com) where 100.000 RBCs were counted. The analysis was performed using FlowJo software. For the erythrocyte binding inhibition assay the same protocol was followed with an additional 30 min pre-incubation of recombinant NTS-DBL1α-domains with different dilutions of the mAbs and pIgGs before incubation with RBCs.

### Biotinylation of mAbs and Competition Assay

mAbs were biotinylated using the EZ-Link NHS-PEG Solid-Phase Biotinylation Kit (Pierce ThermoScientific, product #21440) according to manufacturer’s instructions. Analysis of surface reactivity was carried out as described above with the following modifications: after 30 min incubation with unlabelled mAb (50 ug/ml in 50 µl), pRBC were washed and incubated with biotinylated mAb (50 ug/ml in 50 µl); Streptavidin-FITC (50 µl, dilution 1∶300, Invitrogen) was added for surface staining.

### pIgG Absorption

Peptides were conjugated to NHS activated agarose (Pierce ThermoScientific, product #26197) according to manufacturer’s instructions. Briefly 1 mg/ml (455 µM) of peptide in PBS was mixed with dry resin for 1 hour at RT followed by 30 minutes incubation in Tris pH 8. After re-equilibration of the resin in PBS, 1 ml of pIgG, at 1 mg/ml concentration, was added and incubated overnight at 4°C. After elution the flow-through was re-incubated for 2 hours at RT. The final flow-through was collected, concentrated, and absorption verified by ELISA on the corresponding peptide.

### Peptide Array

Peptide microarrays were manufactured by JPT (***JPT Peptide Technologies,*** Germany) with each slide containing three identical subarrays of ≈100 overlapping, 15aa long, peptides for each of seven NTS-DBL1α-sequences from various parasite laboratory strains and isolates (Table S1). Slides were incubated for 16 hours at 4°C with 5 µg/ml, 250 µl volume, of the antibody of interest in PBS buffer containing 3% of FCS and 0.5% Tween (TPBS). After washing twice with TPBS and trice with distilled water, slides were incubated with species-specific Cy5-conjugated secondary antibody (Jackson ImmunoResearch). Following washing steps, slides were scanned at 635 nm using a GenePix 4000B microarray scanner (Axon Instruments, CA, USA) and images analyzed using GenePixPro 7.0 software in combination with the GAL file provided by JPT. The mean fluorescence intensities obtained from the foreground minus the local background were used to calculate the antibody responses; data presented here correspond to the average of the three subarrays.

### Protein Modeling

The 3D structures of NTS-DBL1α_IT4var60_, NTS-DBL1α_IT4var9_ and NTS-DBL1α-CIDR1γ_IT4var60_ were modeled using the Phyre2 server [54]. The crystal structure of NTS-DBL1α _PAvarO_ (PDB accession code 2XUO) [29] was used as a template. Structural visualizations and high-resolution images were generated using PyMol (The PyMOL Molecular Graphics System, Version 1.3, Schrödinger, LLC.).

### Bioinformatic Analysis

Sequences of 3D7 (52 sequences), IT4 (36 sequences), Dd2 (26 sequences), HB3 (22 sequences) and other parasites (8 sequences) were obtained from GenBank [55], varDB [56] and VarDom [28] databases. The multiple sequence alignments were performed using ClustalW with default parameters and unrooted [57]. Phylogenic trees were built using the Neighbour-Joining method using Molecular Evolutionary Genetic Analysis 5 (MEGA5) [58]. Trees were edited and visualized using Dendroscope [59].

### Statistical Analysis

All values are expressed as mean ± SD. Comparisons between different groups were made using Students unpaired *t* test or Mann-Whitney test (for non-normally distribuited samples). Non-linear regression was used to correlate serum-IgG levels.

## Supporting Information

Figure S1 **Binding of the recombinant NTS-DBL1α domains to RBCs.** Recombinant NTS-DBL1α was incubated at 25, 50 and 100 µg/ml (green, blue and yellow respectively) with RBCs in PBS. Binding was detected with anti-his mAbs, followed by secondary antibody Alexa488-conjugated, by flow cytometry. In red is shown the binding of a control his-tagged recombinant protein (NTS-DBL1α of TM284S2).
(TIF)

Figure S2 **Activity of antibodies towards the NTS-DBL1α-domain of rosette associated PfEMP1 molecules. A:** Surface reactivity of 10 µg/ml pIgG/1∶5 serum, followed by secondary antibody Alexa488-conjugated, with pRBC of the homologous parasite strain/clone as visualized by flow cytometry. nIgG and anti-NTS-DBL1α pIgGs/anti SD3-serum are in red and blue respectively. **B:** Surface reactivity of mAbs (at 20 µg/ml) with homologous pRBC as detected by Alexa488-conjugated secondary antibody and visualized by flow cytometry. mAbs and control mAbSlyD are in blue and red respectively. **C:** Surface reactivity of mAbV2-c20 (at 20 µg/ml), with homologous pRBC as detected by Alexa488-conjugated secondary antibody and visualized by flow cytometry, in presence (+) or absence (–) of human serum proteins. pRBCs were stripped using sodium citrate (5) to remove bound serum proteins (including immunoglobulins) prior to mAb labeling. mAbV2-c20 and control mAbSlyD are in blue and red respectively.
(PDF)

Figure S3 **Results of peptide microarrays. A:** Reactivity of the different mAbs on the NTS-DBL1α_IT4var60_ microarray. The graphs indicate the reactivity of the mAbs towards the 15-mers peptide covering the NTS-DBL1α_IT4var60_ sequence (peptide sequences on the y-axis N-terminal to C-terminal, bottom to top). **B:** Reactivity of the different mAbs on the NTS-DBL1α_IT4var9_ microarray. The graphs indicate the reactivity of the mAbs towards the 15-mers peptide covering the NTS-DBL1α_IT4var9_ sequence (peptide sequences on the y-axis N-terminal to C-terminal, bottom to top). **C:** Reactivity of the pIgGs to IT4var60/FCR3S1.2 on the NTS-DBL1α microarrays. The graphs indicate the reactivity of the pIgG (black line) and nIgG (blue line) towards the 15-mers peptide covering the homologous NTS-DBL1α sequence (peptide sequences on the x-axis N-terminal to C-terminal, left to right). The area corresponding to the SD3-loop is highlighted in red.
(PDF)

Figure S4 **Recognition of SD3-loop by mAbs. A:** Surface labeling competition. Residual surface reactivity of biotinylated (B) mAbs after pre-incubation with unlabeled mAbs. pRBC were pre-incubated with 50 µg/ml unlabeled mAbs and subsequently incubated with 50 µg/ml of biotinylated mAbs. Surface reactivity was detected with Streptavidin-FITC by flow cytometry. Results are shown as residual reactivity relative to biotinylated mAb pre-incubated with PBS. Three different experiments were performed and bars indicate ± SD. **B:** Surface reactivity of biotinylated mAbs with pBRCs, after pre-incubation with unlabelled mAbs, as described under A, visualized by flow cytometry. Plots are representative of typical results showing different degrees of inhibition. Red: mAb SlyD, blue: B-var2–14.1, green: residual reactivity of B-V-14.1 after pre-incubation of pBRCs with mAb as indicated in the figure. **C:** ELISA reactivity of the V2-mAbs towards the SD3-loop peptide (KVKDTCQGYNNSGYRIYCS). ELISA plates were coated with 5 µg/ml of peptide and the reactivity of the mAbs was verified by adding 25 µg/ml of the different mAbs followed by ALP-conjugated secondary antibody. The vertical black bar is the threshold for positivity as calculated by the background binding of mAb-SlyD+2SD.
(TIF)

Figure S5 **Multiple sequence alignment of SD3 sequences. A:** Multiple sequence alignment of SD3 sequences, used to build the phylogenetic tree in Fig. 4, generated using ClustalW. Sequences from helix 6 to helix 7 were aligned. Protein IDs are indicated in the first column while Cys type in the last column (Cys1, Cys2, Cys3, Cys4 or Cys5). 144 unique protein sequences have been used for the alignment and two protein sequences (PFDG_03037 and XP_001351079) have been removed. **B:** Consensus sequence generated from the multiple alignments for the two distinct groups as seen in Fig. 4. The first line indicates the amino acid number, the second line indicates the consensus sequence: aa shaded in black have >99% conservation while aa in grey have >80% consensus. The third line is the motif logo generated using WebLogo.
(PDF)

Figure S6 **Relative localization of the SD3-loop.** Cartoon suggesting possible binding mode of antibodies to the SD3-loop of NTS-DBL1α, maintaining the correct relative sizes of the domains as compared to IgG. The cartoon shows a possible structure of PfEMP1 with the molecular model of NTS-DBL1α-CIDR1γ domains in the N-terminus. The localization of the SD3-loop (red) and an antibody (blue) are indicated.
(TIF)

Table S1 **PfEMP1 sequences on the peptide microarray.**
(DOC)

Table S2 **PCR-primers and vectors used to generate NTSDBL1α-expression constructs.**
(DOC)
