# Supplementary figures and images for: Plasmodium falciparum Rosetting Epitopes Converge in the SD3-Loop of PfEMP1-DBL1α
Source: PLoS One. 2012 Dec 5;7(12):e50758. doi: 10.1371/journal.pone.0050758 (PMC3515580; doi:10.1371/journal.pone.0050758)

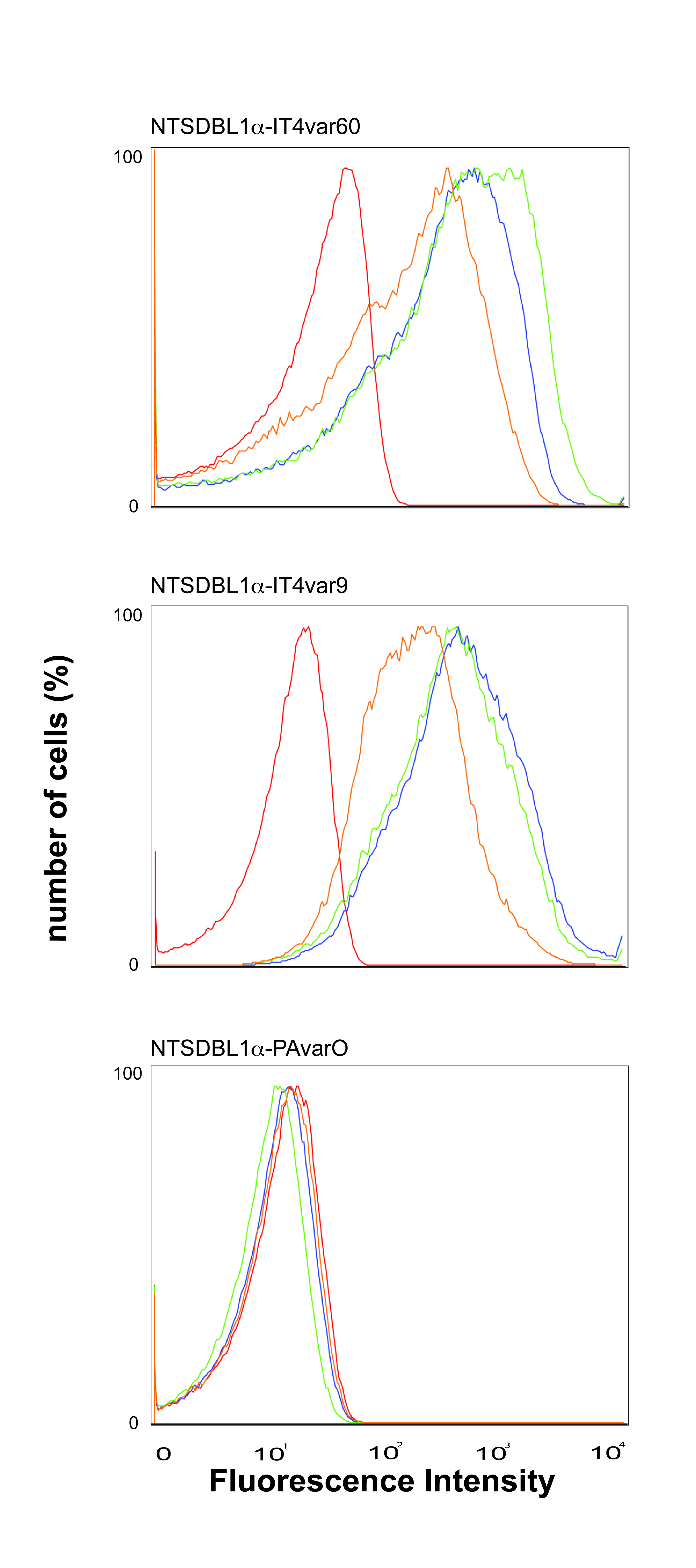

Supplement: Figure S1 — Binding of the recombinant NTS-DBL1α domains to RBCs. Recombinant NTS-DBL1α was incubated at 25, 50 and 100 µg/ml (green, blue and yellow respectively) with RBCs in PBS. Binding was detected with anti-his mAbs, followed by secondary antibody Alexa488-conjugated, by flow cytometry. In red is shown the binding of a control his-tagged recombinant protein (NTS-DBL1α of TM284S2). (TIF) [file pone.0050758.s001.tif]

A

number of cells (%)

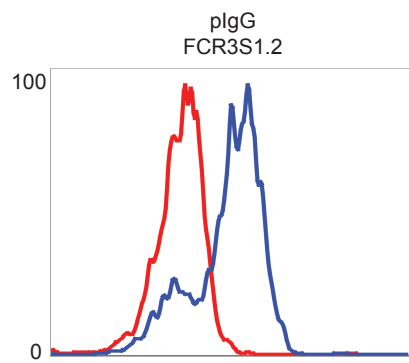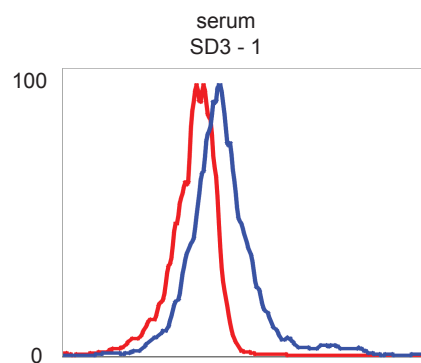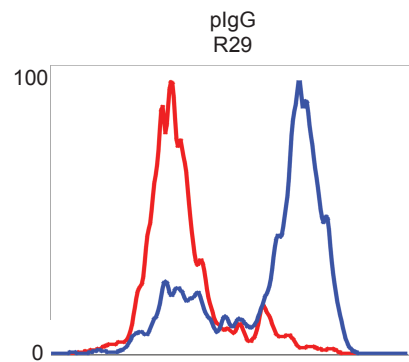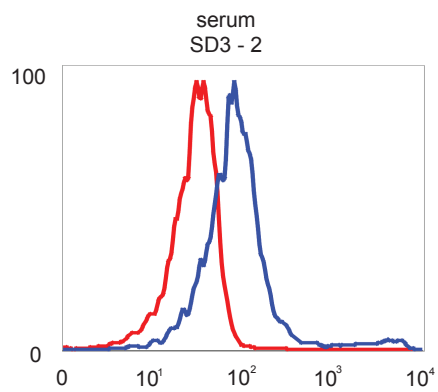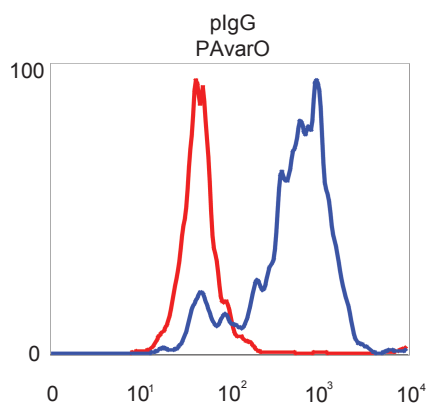

Fluorescence Intensity

B

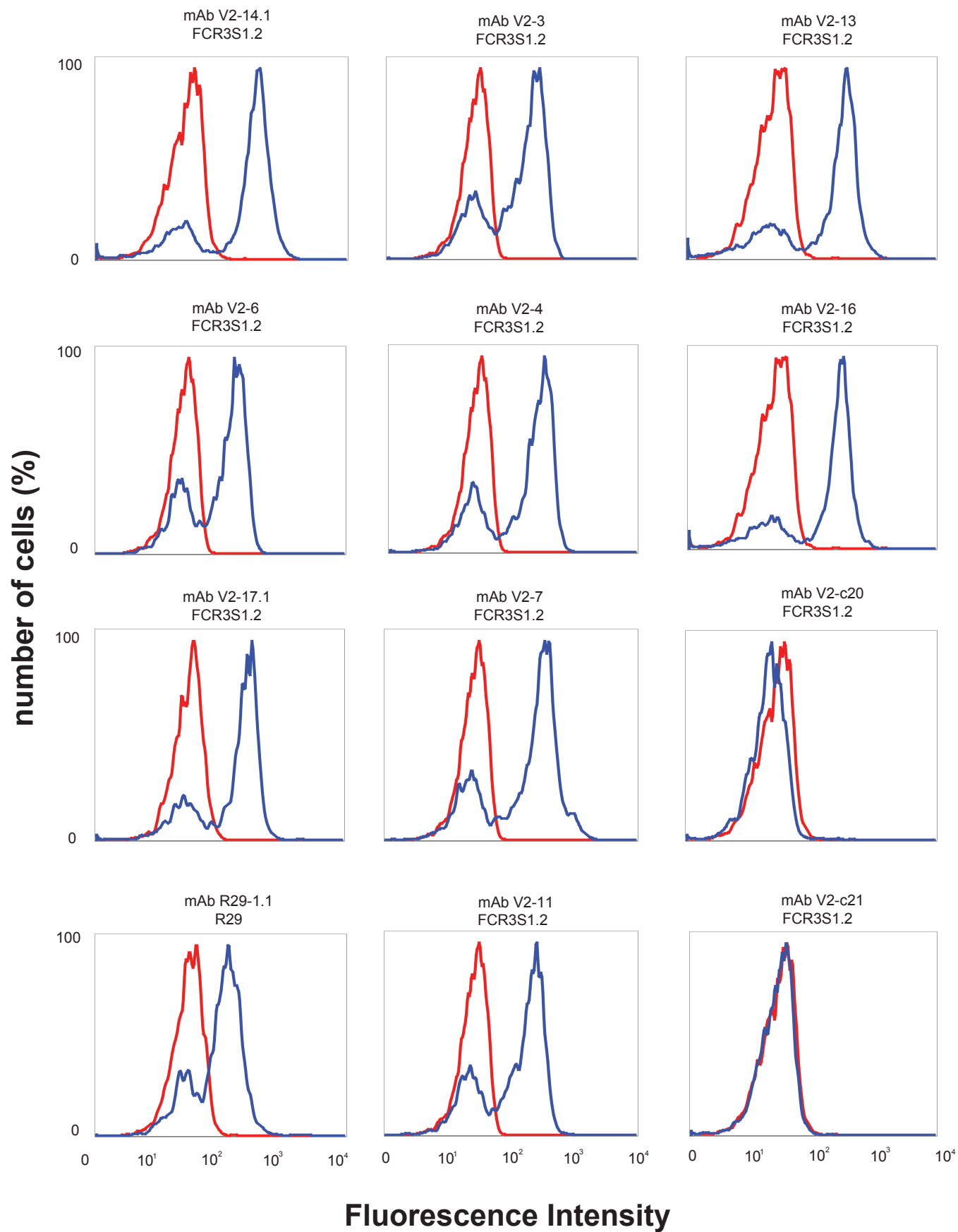

C

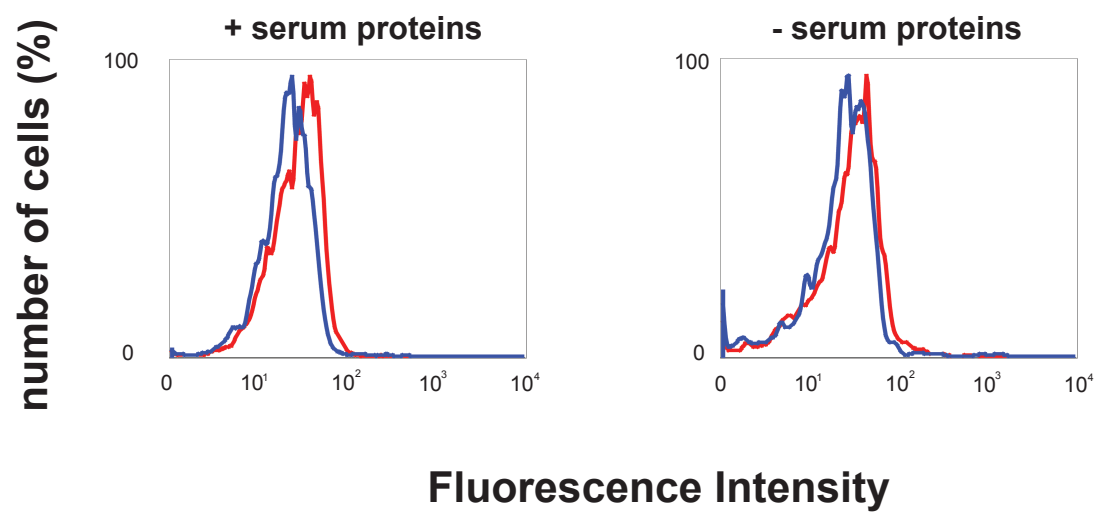

Supplement: Figure S2 — Activity of antibodies towards the NTS-DBL1α-domain of rosette associated PfEMP1 molecules. A: Surface reactivity of 10 µg/ml pIgG/1∶5 serum, followed by secondary antibody Alexa488-conjugated, with pRBC of the homologous parasite strain/clone as visualized by flow cytometry. nIgG and anti-NTS-DBL1α pIgGs/anti SD3-serum are in red and blue respectively. B: Surface reactivity of mAbs (at 20 µg/ml) with homologous pRBC as detected by Alexa488-conjugated secondary antibody and visualized by flow cytometry. mAbs and control mAbSlyD are in blue and red respectively. C: Surface reactivity of mAbV2-c20 (at 20 µg/ml), with homologous pRBC as detected by Alexa488-conjugated secondary antibody and visualized by flow cytometry, in presence (+) or absence (–) of human serum proteins. pRBCs were stripped using sodium citrate (5) to remove bound serum proteins (including immunoglobulins) prior to mAb labeling. mAbV2-c20 and control mAbSlyD are in blue and red respectively. (PDF) [file pone.0050758.s002.pdf]

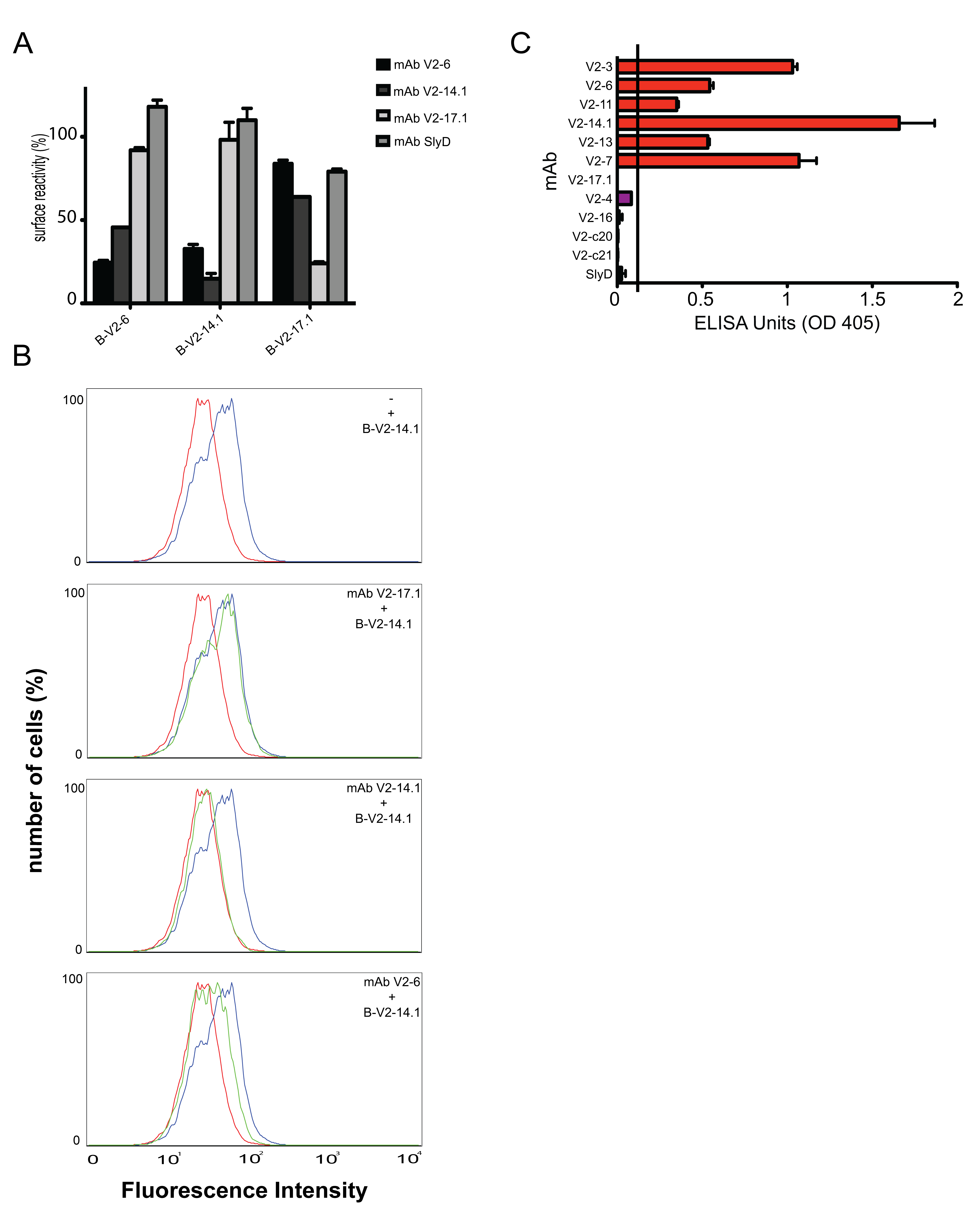

Supplement: Figure S4 — Recognition of SD3-loop by mAbs. A: Surface labeling competition. Residual surface reactivity of biotinylated (B) mAbs after pre-incubation with unlabeled mAbs. pRBC were pre-incubated with 50 µg/ml unlabeled mAbs and subsequently incubated with 50 µg/ml of biotinylated mAbs. Surface reactivity was detected with Streptavidin-FITC by flow cytometry. Results are shown as residual reactivity relative to biotinylated mAb pre-incubated with PBS. Three different experiments were performed and bars indicate ± SD. B: Surface reactivity of biotinylated mAbs with pBRCs, after pre-incubation with unlabelled mAbs, as described under A, visualized by flow cytometry. Plots are representative of typical results showing different degrees of inhibition. Red: mAb SlyD, blue: B-var2–14.1, green: residual reactivity of B-V-14.1 after pre-incubation of pBRCs with mAb as indicated in the figure. C: ELISA reactivity of the V2-mAbs towards the SD3-loop peptide (KVKDTCQGYNNSGYRIYCS). ELISA plates were coated with 5 µg/ml of peptide and the reactivity of the mAbs was verified by adding 25 µg/ml of the different mAbs followed by ALP-conjugated secondary antibody. The vertical black bar is the threshold for positivity as calculated by the background binding of mAb-SlyD+2SD. (TIF) [file pone.0050758.s004.tif]

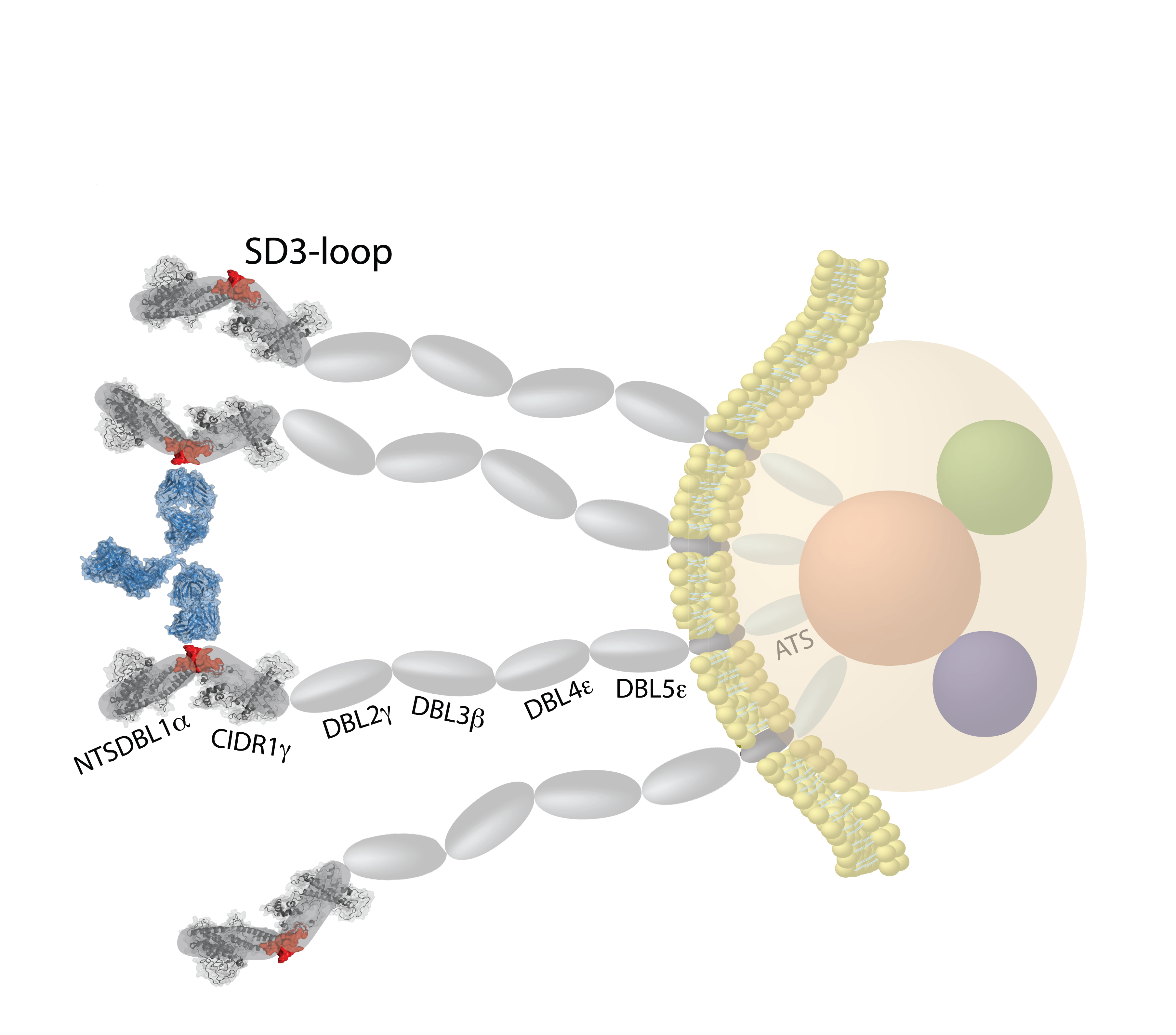

Supplement: Figure S6 — Relative localization of the SD3-loop. Cartoon suggesting possible binding mode of antibodies to the SD3-loop of NTS-DBL1α, maintaining the correct relative sizes of the domains as compared to IgG. The cartoon shows a possible structure of PfEMP1 with the molecular model of NTS-DBL1α-CIDR1γ domains in the N-terminus. The localization of the SD3-loop (red) and an antibody (blue) are indicated. (TIF) [file pone.0050758.s006.tif]
