# Supplementary material for: Plasmodium falciparum Rosetting Epitopes Converge in the SD3-Loop of PfEMP1-DBL1α
Source: PLoS One. 2012 Dec 5;7(12):e50758. doi: 10.1371/journal.pone.0050758 (PMC3515580; doi:10.1371/journal.pone.0050758)
Supplement: Figure S3 — Results of peptide microarrays. A: Reactivity of the different mAbs on the NTS-DBL1αIT4var60 microarray. The graphs indicate the reactivity of the mAbs towards the 15-mers peptide covering the NTS-DBL1αIT4var60 sequence (peptide sequences on the y-axis N-terminal to C-terminal, bottom to top). B: Reactivity of the different mAbs on the NTS-DBL1αIT4var9 microarray. The graphs indicate the reactivity of the mAbs towards the 15-mers peptide covering the NTS-DBL1αIT4var9 sequence (peptide sequences on the y-axis N-terminal to C-terminal, bottom to top). C: Reactivity of the pIgGs to IT4var60/FCR3S1.2 on the NTS-DBL1α microarrays. The graphs indicate the reactivity of the pIgG (black line) and nIgG (blue line) towards the 15-mers peptide covering the homologous NTS-DBL1α sequence (peptide sequences on the x-axis N-terminal to C-terminal, left to right). The area corresponding to the SD3-loop is highlighted in red. (PDF) [file pone.0050758.s003.pdf]

A

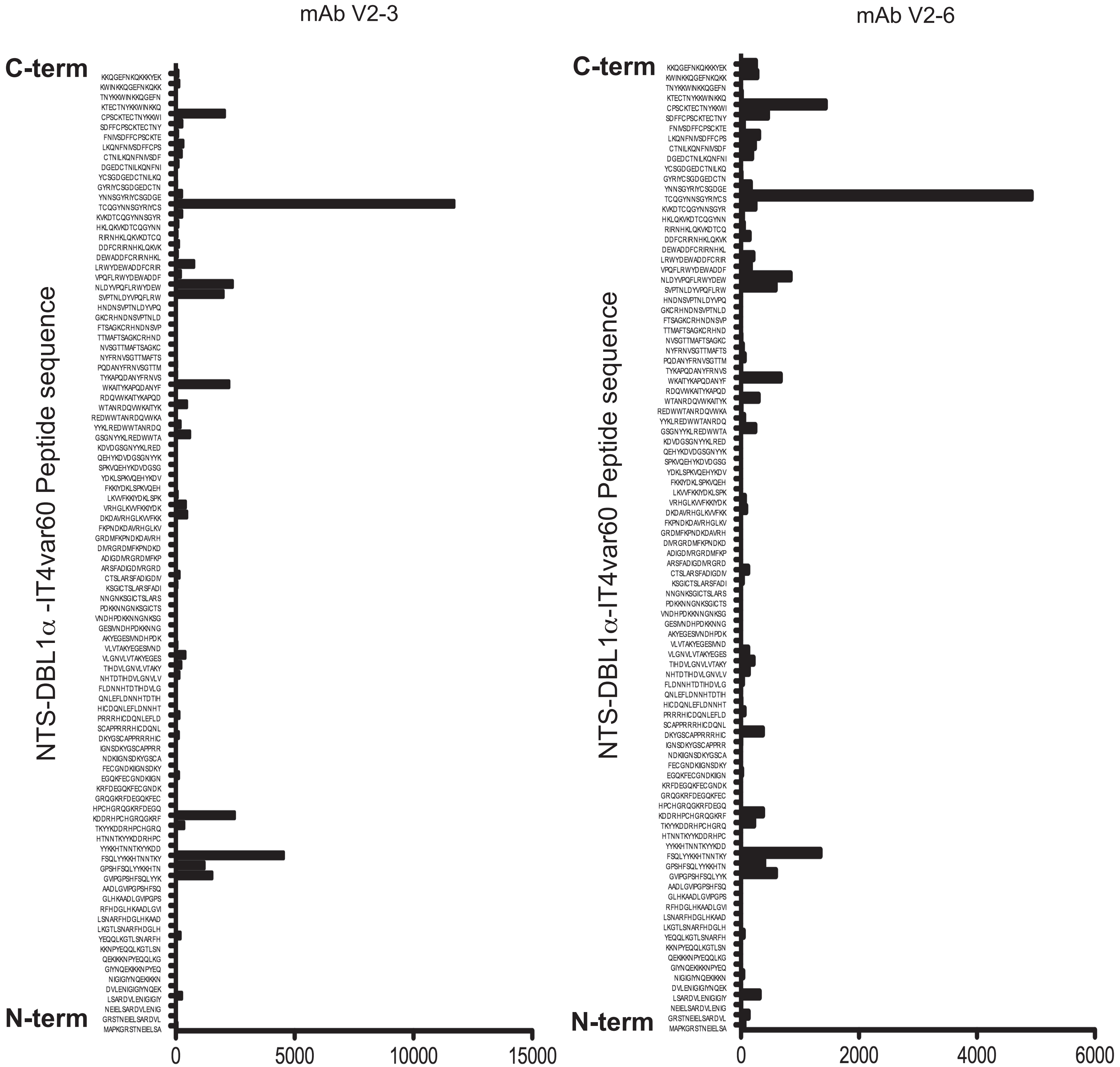
mAb V2-3
mAb V2-6
C-term
N-term
NTS-DBL1α -IT4var60 Peptide sequence
0
5000
10000
15000
2000
4000
6000
Antibody reactivity (AU)

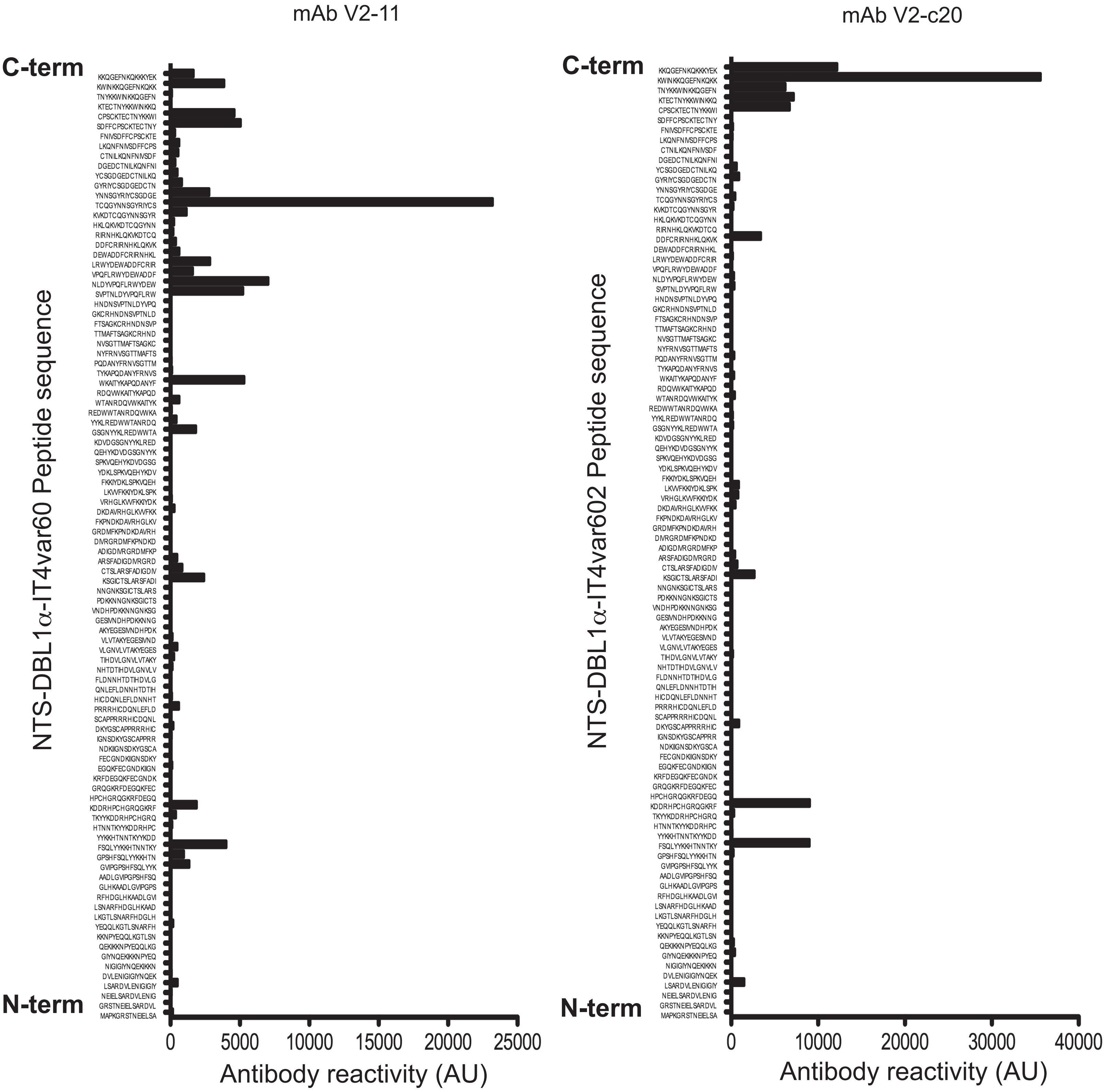
mAb V2-11
mAb V2-c20
C-term
N-term
NTS-DBL1α-IT4var60 Peptide sequence
NTS-DBL1α-IT4var602 Peptide sequence
0
5000
10000
15000
20000
25000
0
10000
20000
30000
40000
Antibody reactivity (AU)
Antibody reactivity (AU)

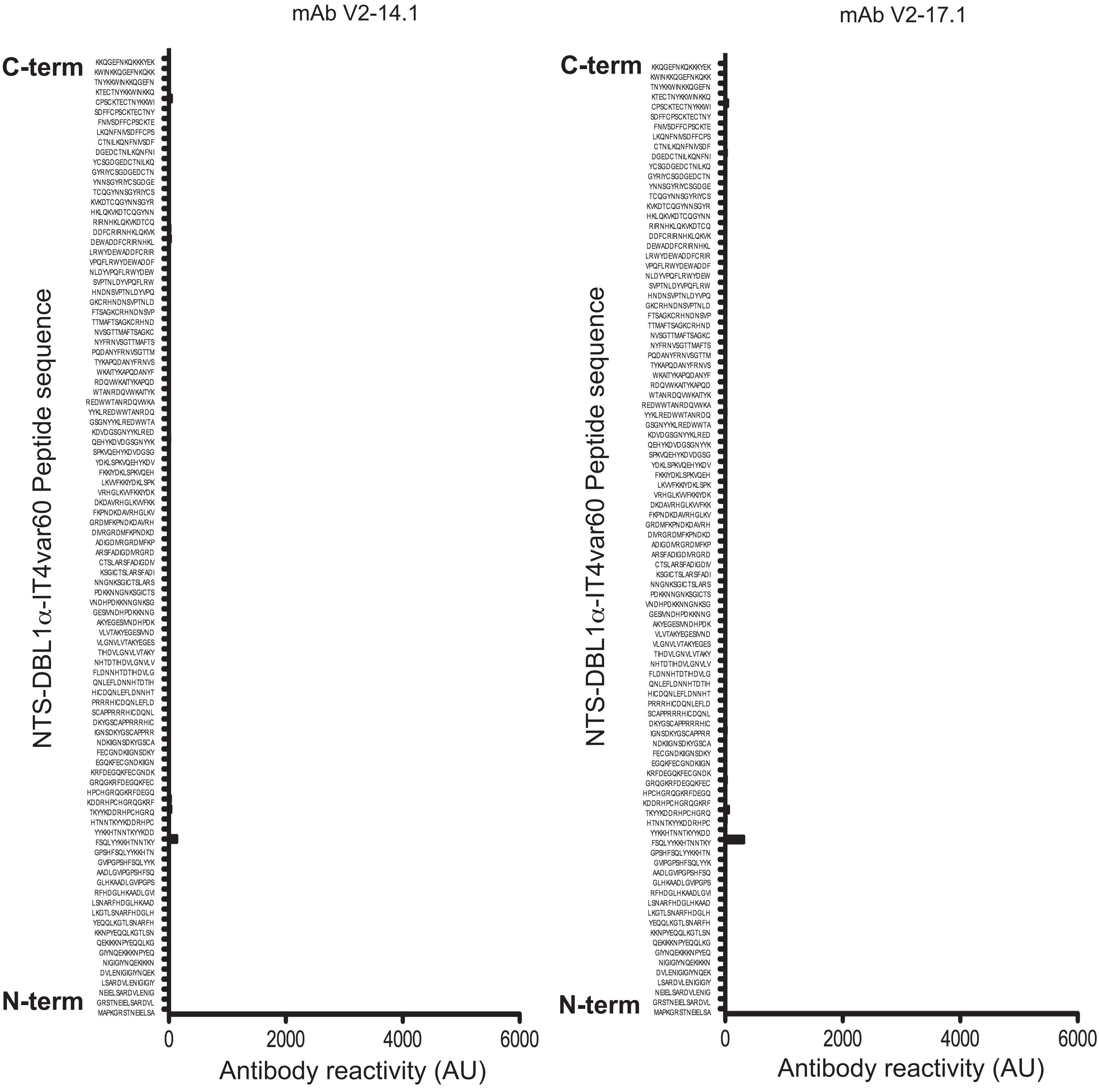

mAb V2-14.1
mAb V2-17.1
C-term
N-term
NTS-DBL1α-IT4var60 Peptide sequence
0
2000
4000
6000
Antibody reactivity (AU)

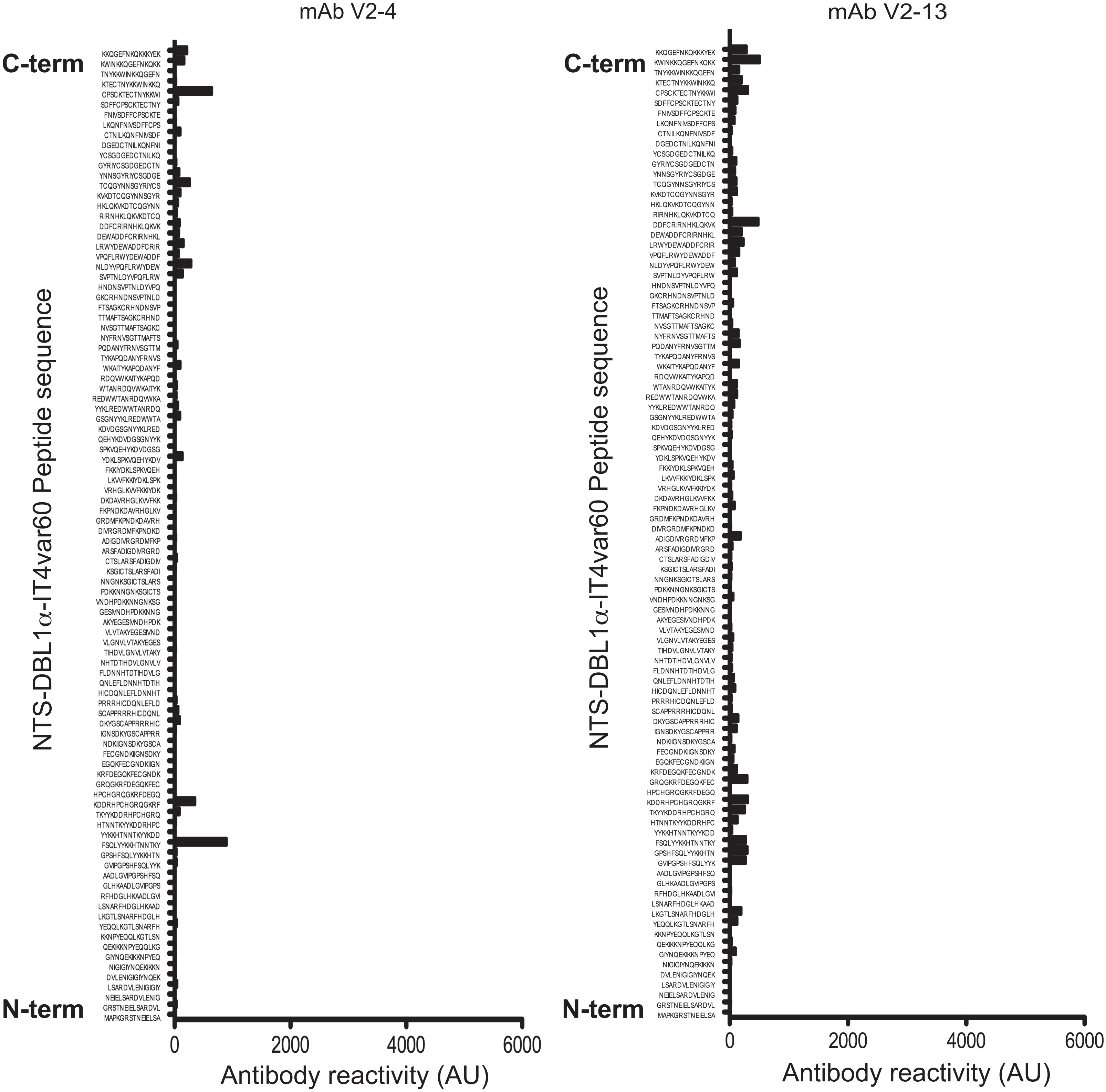
mAb V2-4
mAb V2-13
C-term
N-term
NTS-DBL1α-IT4var60 Peptide sequence
0
2000
4000
6000
Antibody reactivity (AU)

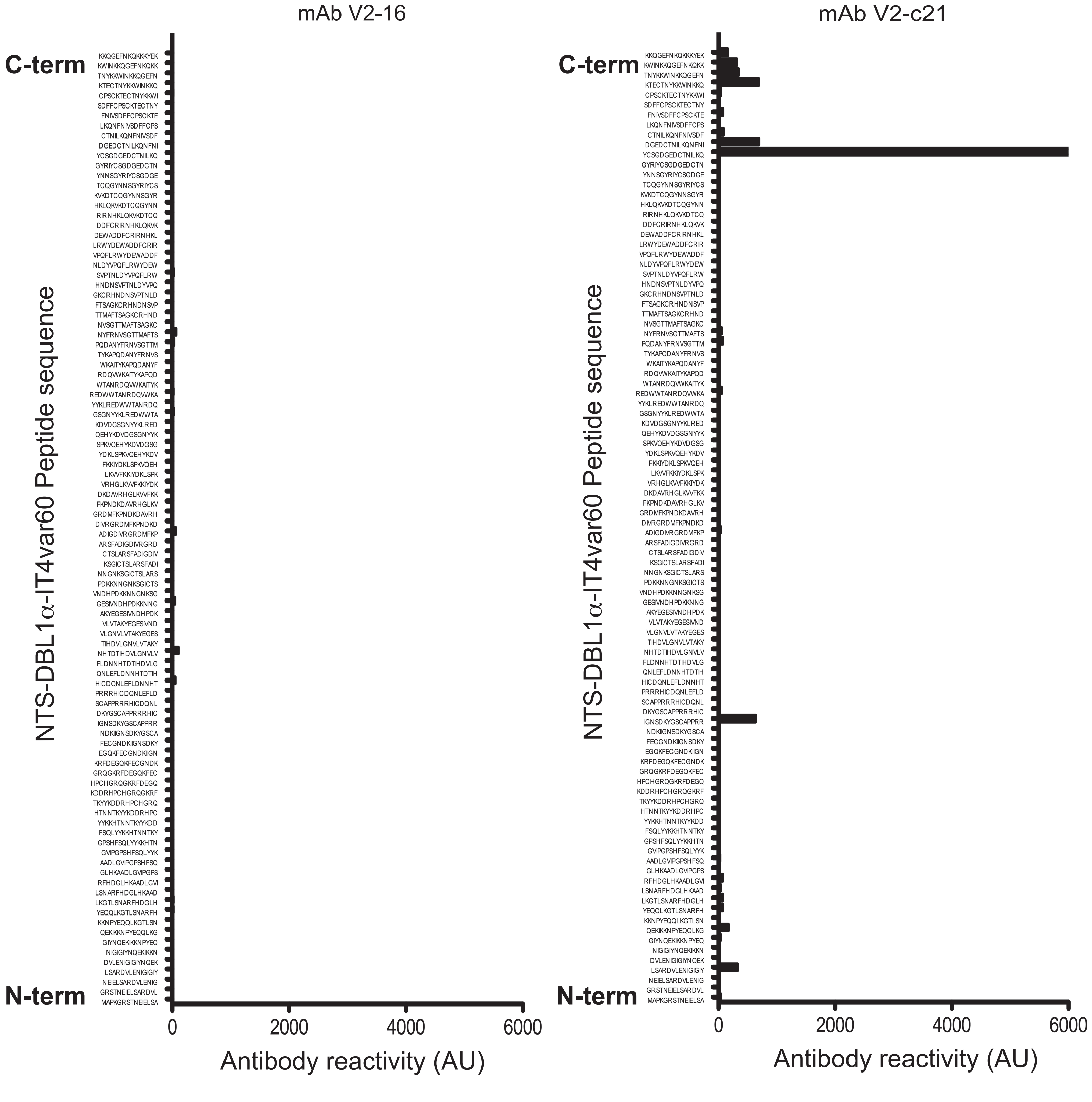

mAb V2-16
mAb V2-c21
C-term
N-term
NTS-DBL1α-IT4var60 Peptide sequence
0
2000
4000
6000
Antibody reactivity (AU)

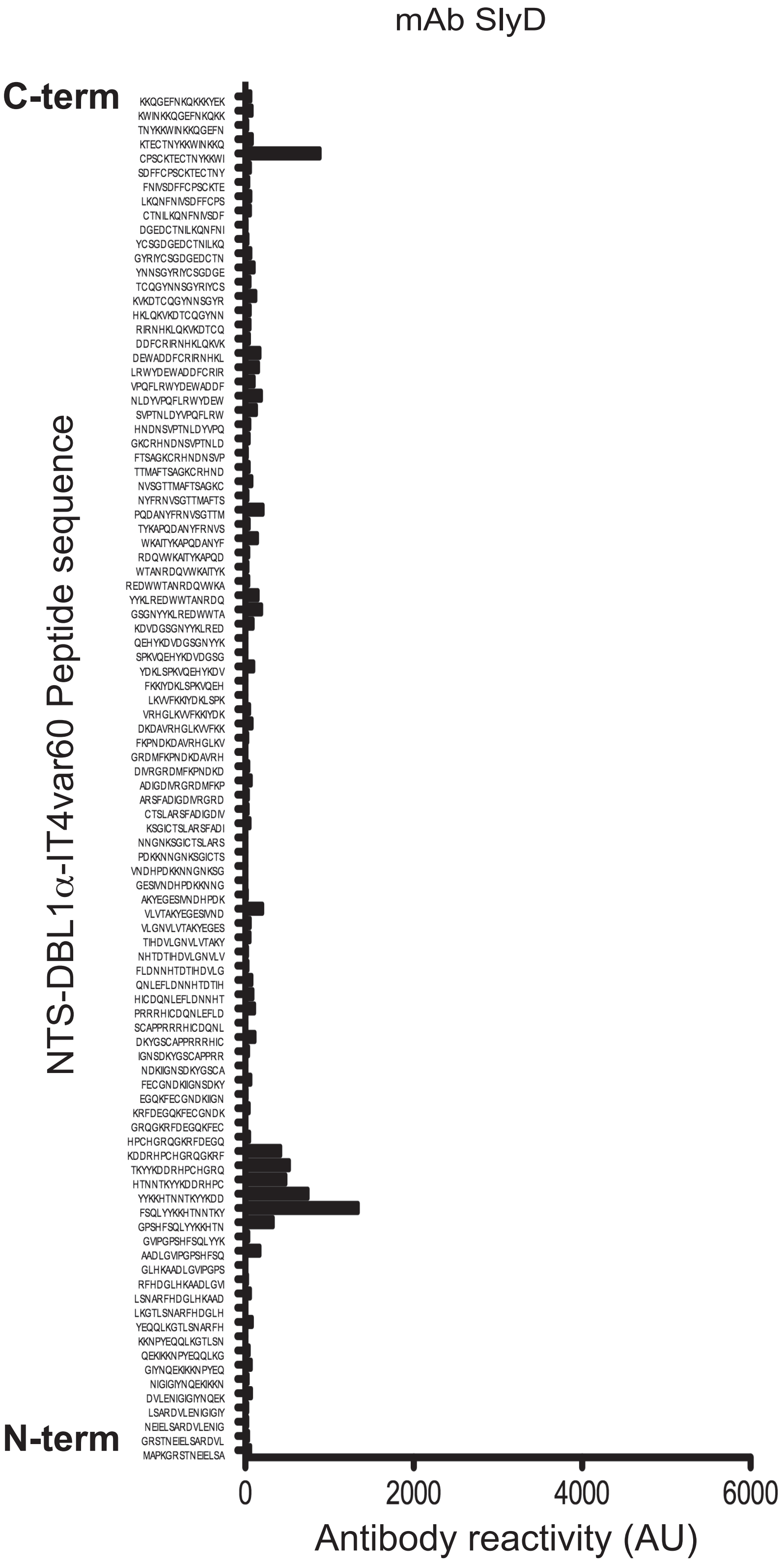
mAb SlyD
C-term
N-term
NTS-DBL1α-IT4var60 Peptide sequence
KKQGEFNKQKKKYEK
KWINKKQGEFNKQKK
TNYKKWINKKQGEFN
KTECTNYKKWINKKQ
CPSCKTECTNYKKWI
SDFFCPSCKTECTNY
FNIVSDFFCPSCKTE
LKQNFNIVSDFFCPS
CTNILKQNFNIVSDF
DGEDCTNILKQNFNI
YCSGDGEDCTNILKQ
GYRIYCSGDGEDCTN
YNNSGYRIYCSGDGE
TCQGYNNSGYRIYCS
KVKDTCQGYNNSGYR
HKLQKVKDTCQGYNN
RIRNHKLQKVKDTCQ
DDFCRIRNHKLQKVK
DEWADDFCRIRNHKL
LRWYDEWADDFCRIR
VPQFLRWYDEWADDF
NLDYVPQFLRWYDEW
SVPTNLDYVPQFLRW
HNDNSVPTNLDYVPQ
GKCRHNDNSVPTNLD
FTSAGKCRHNDNSVP
TTMAFTSAGKCRHND
NVSGTTMAFTSAGKC
NYFRNVSGTTMAFTS
PQDANYFRNVSGTTM
TYKAPQDANYFRNVS
WKAITYKAPQDANYF
RDQVWKAITYKAPQD
WTANRDQVWKAITYK
REDWWTANRDQVWKA
YYKLREDWWTANRDQ
GSGNYYKLREDWWTA
KDVDGSGNYYKLRED
QEHYKDVDGSGNYYK
SPKVQEHYKDVDGSG
YDKLSPKVQEHYKDV
FKKIYDKLSPKVQEH
LKVVFKKIYDKLSPK
VRHGLKVVFKKIYDK
DKDAVRHGLKVVFKK
FKPNDKDAVRHGLKV
GRDMFKPNDKDAVRH
DIVRGRDMFKPNDKD
ADIGDIVRGRDMFKP
ARSFADIGDIVRGRD
CTSLARSFADIGDIV
KSGICTSLARSFADI
NNGNKSGICTSLARS
PDKKNNGNKSGICTS
VNDHPDKKNNGNKSG
GESIVNDHPDKKNNG
AKYEGESIVNDHPDK
VLVTAKYEGESIVND
VLGNVLVTAKYEGES
TIHDVLGNVLVTAKY
NHTDTIHDVLGNVLV
FLDNNHTDTIHDVLG
QNLEFLDNNHTDTIH
HICDQNLEFLDNNHT
PRRRHICDQNLEFLD
SCAPPRRRHICDQNL
DKYGSCAPPRRRHIC
IGNSDKYGSCAPPRR
NDKIIGNSDKYGSCA
FECGNDKIIGNSDKY
EGQKFECGNDKIIGN
KRFDEGQKFECGNDK
GRQGKRFDEGQKFEC
HPCHGRQGKRFDEGQ
KDDRHPCHGRQGKRF
TKYYKDDRHPCHGRQ
HTNNTKYYKDDRHPC
YYKKHTNNTKYYKDD
FSQLYYKKHTNNTKY
GPSHFSQLYYKKHTN
GVIPGPSHFSQLYYK
AADLGVIPGPSHFSQ
GLHKAADLGVIPGPS
RFHDGLHKAADLGVI
LSNARFHDGLHKAAD
LKGTLSNARFHDGLH
YEQQLKGTLSNARFH
KKNPYEQQLKGTLSN
QEKIKKNPYEQQLKG
GIYNQEKIKKNPYEQ
NIGIGIYNQEKIKKN
DVLENIGIGIYNQEK
LSARDVLENIGIGIY
NEIELSARDVLENIG
GRSTNEIELSARDVL
MAPKGRSTNEIELSA
0
2000
4000
6000
Antibody reactivity (AU)

B

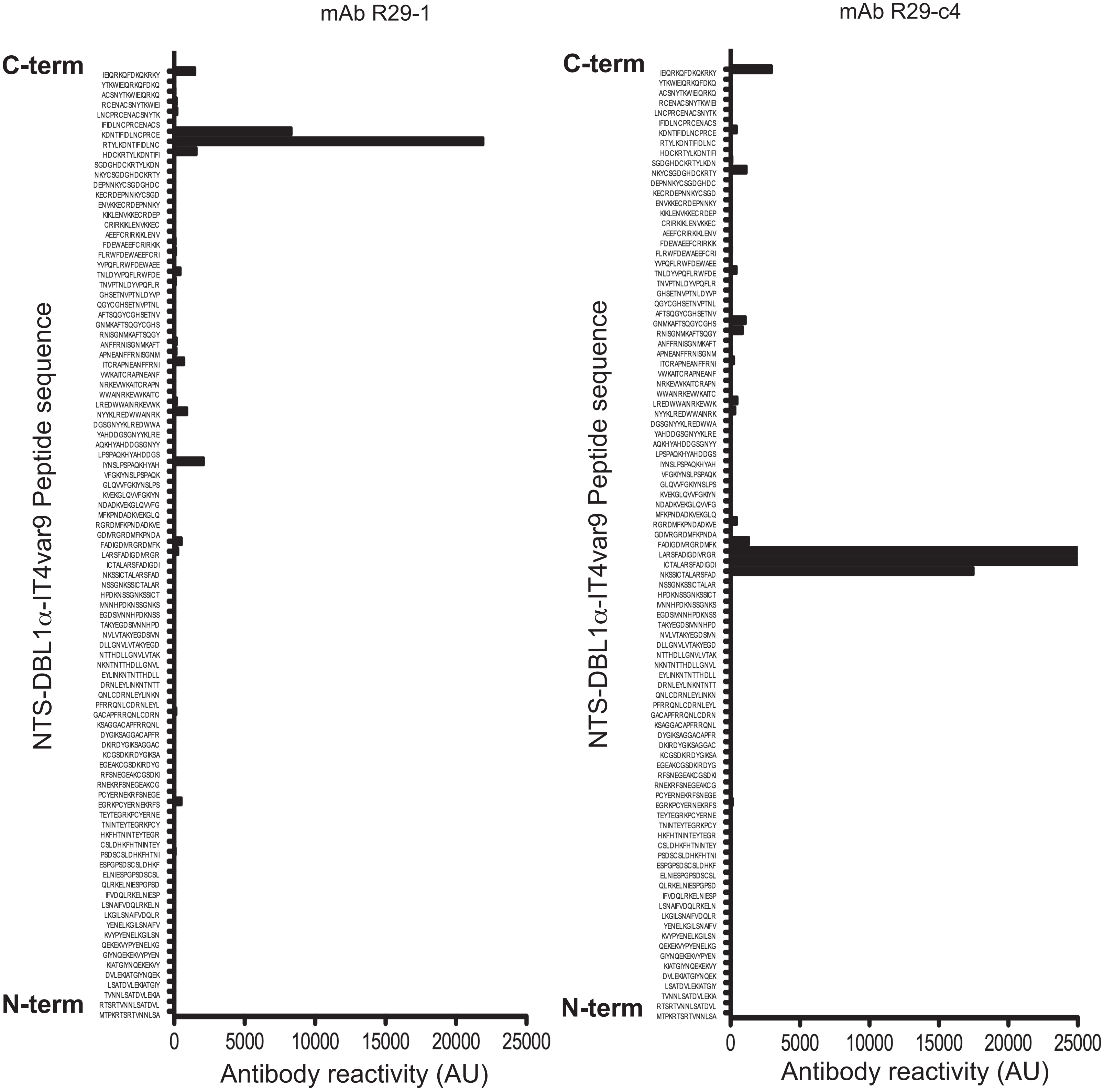
mAb R29-1
mAb R29-c4
C-term
N-term
NTS-DBL1α-IT4var9 Peptide sequence
0
5000
10000
15000
20000
25000
Antibody reactivity (AU)

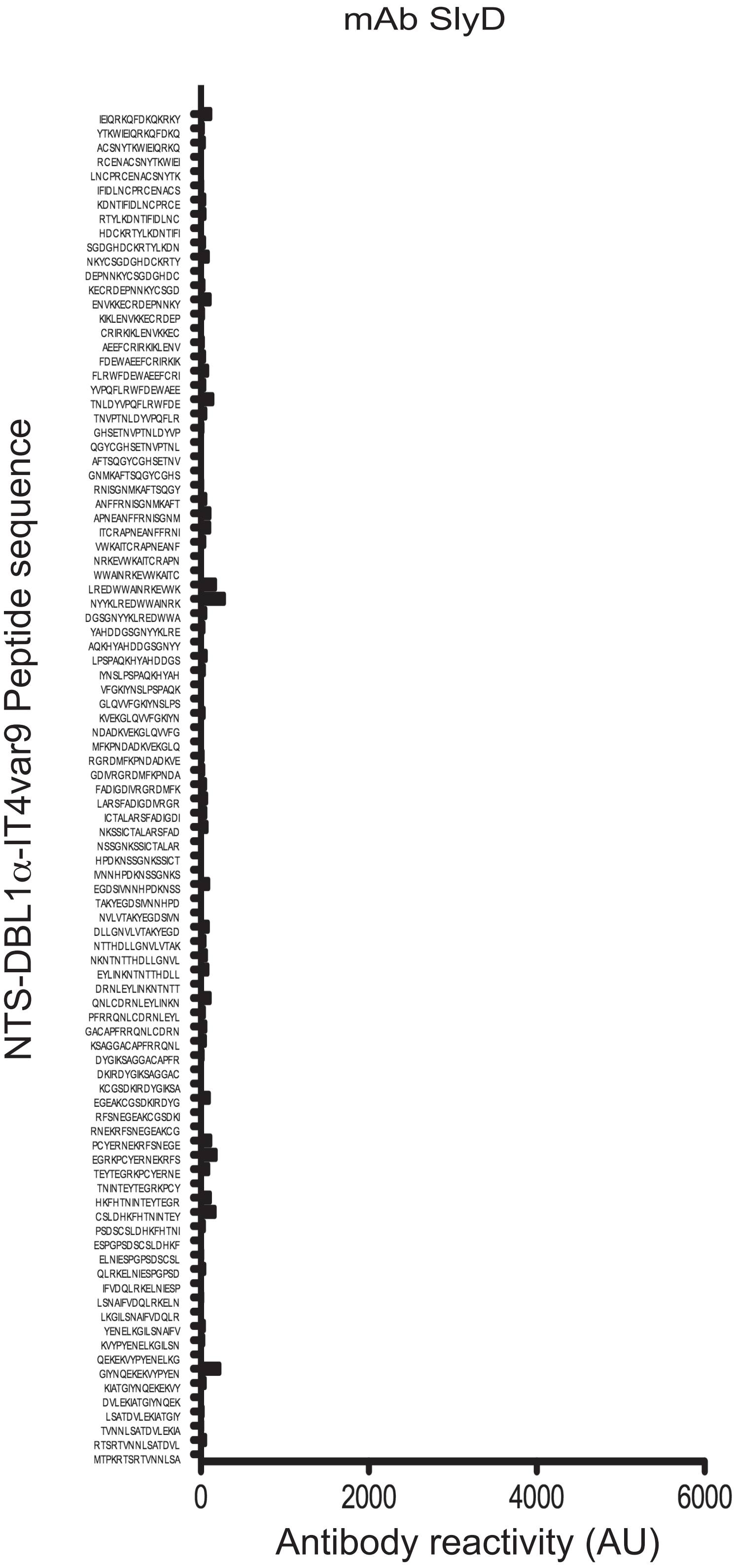
mAb SlyD
NTS-DBL1α-IT4var9 Peptide sequence
0
2000
4000
6000
Antibody reactivity (AU)

C

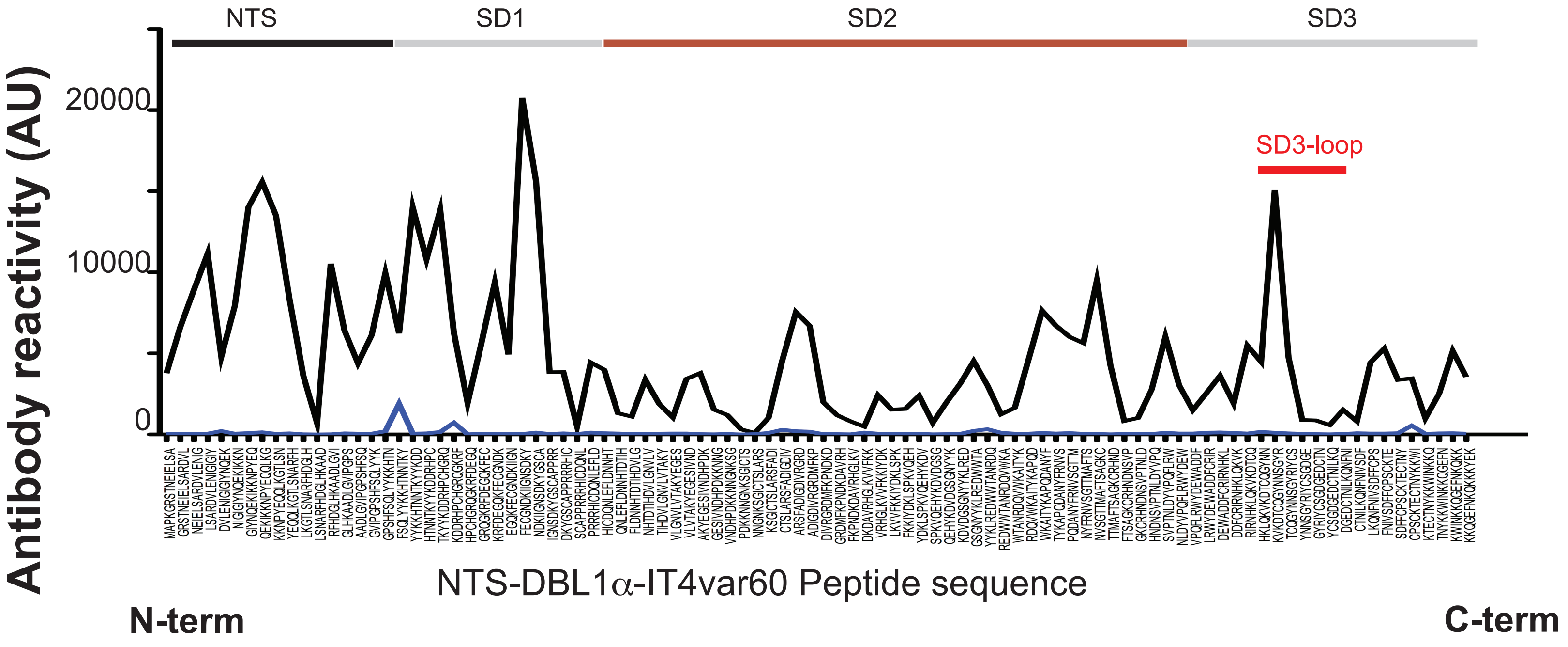
NTS
SD1
SD2
SD3
SD3-loop
Antibody reactivity (AU)
20000
10000
0
NTS-DBL1α-IT4var60 Peptide sequence
N-term
C-term
