# Supplementary material for: Plasmodium falciparum Rosetting Epitopes Converge in the SD3-Loop of PfEMP1-DBL1α
Source: PLoS One. 2012 Dec 5;7(12):e50758. doi: 10.1371/journal.pone.0050758 (PMC3515580; doi:10.1371/journal.pone.0050758)
Supplement: Figure S5 — Multiple sequence alignment of SD3 sequences. A: Multiple sequence alignment of SD3 sequences, used to build the phylogenetic tree in Fig. 4, generated using ClustalW. Sequences from helix 6 to helix 7 were aligned. Protein IDs are indicated in the first column while Cys type in the last column (Cys1, Cys2, Cys3, Cys4 or Cys5). 144 unique protein sequences have been used for the alignment and two protein sequences (PFDG_03037 and XP_001351079) have been removed. B: Consensus sequence generated from the multiple alignments for the two distinct groups as seen in Fig. 4. The first line indicates the amino acid number, the second line indicates the consensus sequence: aa shaded in black have >99% conservation while aa in grey have >80% consensus. The third line is the motif logo generated using WebLogo. (PDF) [file pone.0050758.s005.pdf]

A

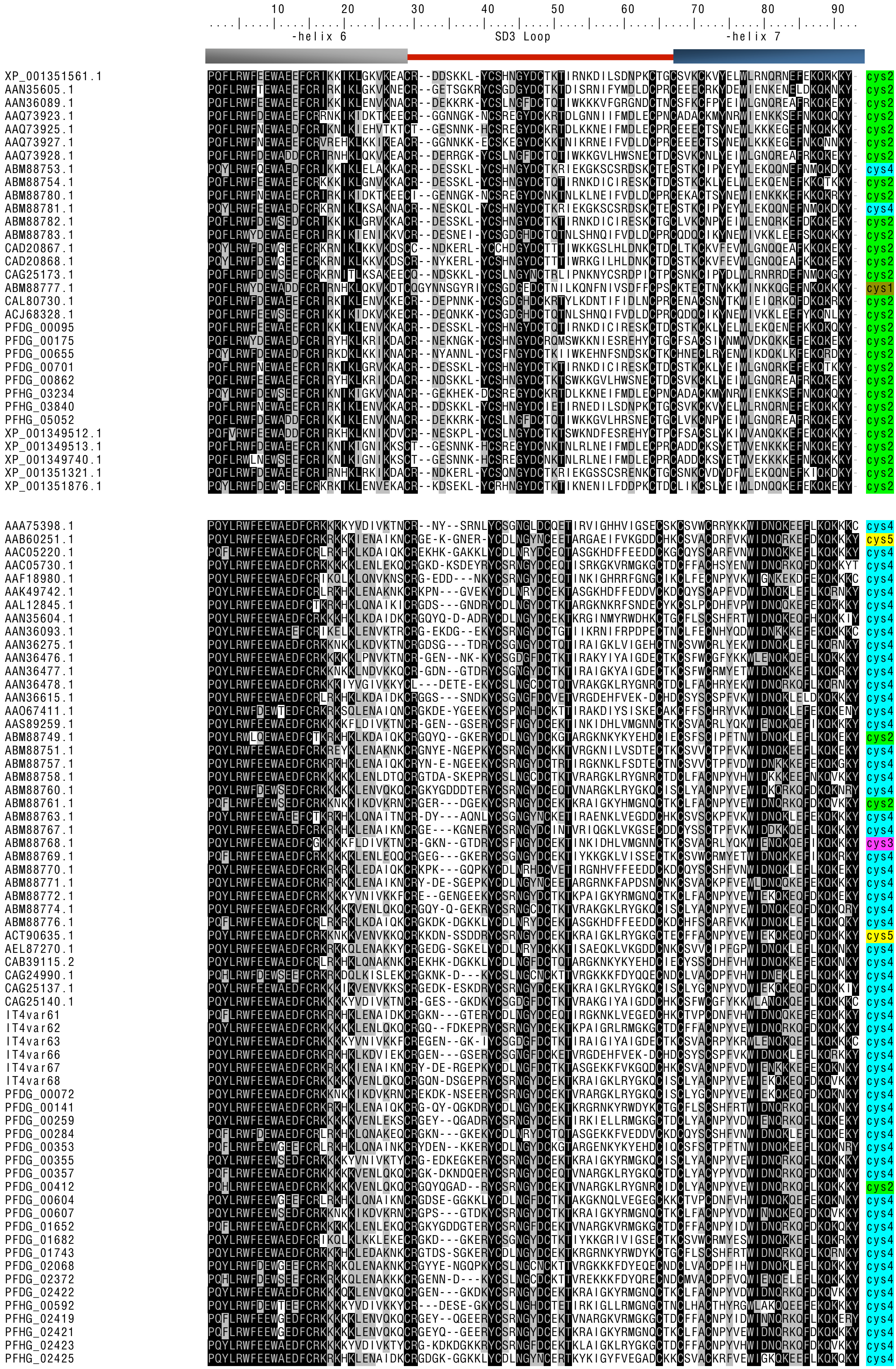
10 20 30 40 50 60 70 80 90
-helix 6
SD3 Loop
-helix 7
XP_001351561.1 cys2
AAN35605.1 cys2
AAN36089.1 cys2
AAQ73923.1 cys2
AAQ73925.1 cys2
AAQ73927.1 cys2
AAQ73928.1 cys2
ABM88753.1 cys4
ABM88754.1 cys2
ABM88780.1 cys2
ABM88781.1 cys4
ABM88782.1 cys2
ABM88783.1 cys2
CAD20867.1 cys2
CAD20868.1 cys2
CAG25173.1 cys2
ABM88777.1 cys1
CAL80730.1 cys2
ACJ68328.1 cys2
PFDG_00095 cys2
PFDG_00175 cys2
PFDG_00655 cys2
PFDG_00701 cys2
PFDG_00862 cys2
PFHG_03234 cys2
PFHG_03840 cys2
PFHG_05052 cys2
XP_001349512.1 cys2
XP_001349513.1 cys2
XP_001349740.1 cys2
XP_001351321.1 cys2
XP_001351876.1 cys2
AAA75398.1 cys4
AAB60251.1 cys5
AAC05220.1 cys4
AAC05730.1 cys4
AAF18980.1 cys4
AAK49742.1 cys4
AAL12845.1 cys4
AAN35604.1 cys4
AAN36093.1 cys4
AAN36275.1 cys4
AAN36476.1 cys4
AAN36477.1 cys4
AAN36478.1 cys4
AAN36615.1 cys4
AAO67411.1 cys4
AAS89259.1 cys4
ABM88749.1 cys2
ABM88751.1 cys4
ABM88757.1 cys4
ABM88758.1 cys4
ABM88760.1 cys4
ABM88761.1 cys2
ABM88763.1 cys4
ABM88767.1 cys4
ABM88768.1 cys3
ABM88769.1 cys4
ABM88770.1 cys4
ABM88771.1 cys4
ABM88772.1 cys4
ABM88774.1 cys4
ABM88776.1 cys4
ACT90635.1 cys5
AEL87270.1 cys4
CAB39115.2 cys4
CAG24990.1 cys4
CAG25137.1 cys4
CAG25140.1 cys4
IT4var61 cys4
IT4var62 cys4
IT4var63 cys4
IT4var66 cys4
IT4var67 cys4
IT4var68 cys4
PFDG_00072 cys4
PFDG_00141 cys4
PFDG_00259 cys4
PFDG_00284 cys4
PFDG_00353 cys4
PFDG_00355 cys4
PFDG_00357 cys4
PFDG_00412 cys2
PFDG_00604 cys4
PFDG_00607 cys4
PFDG_01652 cys4
PFDG_01682 cys4
PFDG_01743 cys4
PFDG_02068 cys4
PFDG_02372 cys4
PFDG_02422 cys4
PFHG_00592 cys4
PFHG_02419 cys4
PFHG_02421 cys4
PFHG_02423 cys4
PFHG_02425 cys4

```
                    10        20        30        40        50        60        70        80        90
           ....|....|....|....|....|....|....|....|....|....|....|....|....|....|....|....|....|....|...
                 α-helix 6                   SD3 Loop                          α-helix 7

PFHG_02429      PQYLRWLHEWAEDFCRKKNKKIKDVKRNCR--GP-SGTDKYCSRNGYDCEKTVRARGKLRYGNRCIDCLYACNPYVHWIDKKKEEFDKQKKKY  cys4
PFHG_03232      PQFLRWFDEWAEDFCRKRKHKLQNAIKNCR--TP-NGKDKYCDLNGYNCEETAKKENKLFPDSECHKCSVACNPFVPWIDNQKKEFEKQKGKY  cys4
PFHG_03416      PQYLRWFEEWAEDFCRKKKKKIPNVKTNCRQVKE-S-KEKYCTVGGYNCDGTIRKQYIYRLDTDCTKCSLACKTFAEWIDNQKQEFLKQKQKY  cys4
PFHG_03480      PQYLRWFEEWAEDFCRKKNKKIKDVQKQCRGTDS-SGKKRYCSRNGYDCEKTKRAIGKYRMGNQCISCLYGCNPYVEWIDNQRKQFDKQVKKY  cys4
PFHG_03516      PQYLRWFEEWAEDFCRKKNKKLEDAIKKCRGQDG-SGEKRYCDLNGYDCKRTFSAKKKYRWDHKCTGCFLSCSHFRTWIDNQKEQFLKQKNKY  cys4
PFHG_04014      PQFLRWFEEWAEDFCRKKAKKLQNAIKNCRGPN---GNDKYCSRNGYDCTKTKRAIGKYRMGNQCTKCLFACNPYVEWIDKKKEEFDKQVKKY  cys2
PFHG_04015      PQYLRWFDEWSEDFCRKKKKKLENVQKQCRGKYGDDDTERYCSGNGYDCERTIYKKGKLVIGYQCTNCSVLCRIYEKWIDNQKEQFLKQKGKY  cys4
PFHG_04368      PQYLRWFEEWAEDFCRKKKKKVENLEQQCRGKSEE-GEPRYCDRNGFDCERTKYKKGYFVIDKGCNTCSVWCRLYESWIDNQKKEFLKQKKKC  cys4
PFHG_04491      PQYLRWFEEWAEDFCRLRKHKLQNAKSKCRGEKN-D---KYCSRNGCDCTKTVRGKNKFSYEHECNDCLVACDPFVHWIDNQKLEFLKQKNKY  cys4
PFHG_04593      PQYLRWFDEWGEEFCRKRKKQLENAKKKCRGQDK-EGKERYCDLNGYDCEKTKRGRNMYRWDYKCTGCFLSCSHFRTWIDNQKEQFLKQRNKY  cys4
PFHG_04749      PQYLRWLQEWAEDFCRKKKIKVENVKKSCRGEN---GKDKYCSRNGYDCTKTKRAIGKLRYGNRCIDCLYACNPYVHWIDNQKEQFDKQVKKY  cys4
PFHG_04770      PQYLRWFEEWAEDFCRLRKHKLKDAIKKCRGDN---GTERYCDLNRHDCTKTASGKHDFFEGDDCIGCHFSCSHFVKWIDNQKLEFLKQKKKC  cys4
PFHG_04910      PQYLRWFQEWAEEFCRKKKKYVNIVKKYCR---DEKE-GKYCSLNGHDCTQTIRKIGLLRMGNECTKCLHACSHYRGWLANQKKEFEKQKEKY  cys2
PFHG_05400      PQYLRWFDEWTEEFCRLRRKKIEILEKICRG-KNDSGQPKYCSRNGCDCEQTINKIGRIRLGNGCTNCLFACNRYIDWINNKKKEFLKQKKKY  cys4
XP_001347288.1  PQYLRWFEEWAEDFCRKKKIYVDIVKKYCR---GERDGEKYCSLNGCDCTKTVRARGKLRYGNRCTDCLFACHRYENWIDNQRKQFLKQRNKY  cys5
XP_001347690.2  PQYLRWFEEWAEEFCRLRKHKLQNAIKNCRGEN----NEKYCDLNGYDCEKTIRGKKKLFEGADCKKCTVTCDNFVPWIKNQKQEFDKQKNKY  cys4
XP_001349030.1  PQYLRWFEEWAEDFCRKKKKKVQNLQKQCRDKY--QGDDRYCSRNGYDCEQTINKIGKLVIGKGCINCLYACNPYVDWINNQKEQFDKQVKKY  cys4
XP_001349031.1  PQYIRWFEEWAEDFCRKKKKKVQNLQKQCRGTD-ASKEPRYCSRNGFDCEKTISRIGKVRMGKGCTDCFFACHRYENWIDNQRKQFLKQKNIY  cys4
XP_001349032.1  PQYLRWFEEWAEDFCRKKKKYVDIVKTYCRG-KYQ-GEERYCSRNGFDCEKTVNARGKVRMGKGCTDCFFACNPYVDWIDNQRKQFLKQRNKY  cys4
XP_001349033.1  PQYLRWFEEWAEEFCRKRKKKLQNAQKFCR--DE-SSK-LYCSLNGYDCKATIRANDEYTISQACAKCFFSCHRFVKWIDNQKLELEKQKKKY  cys3
XP_001349034.1  PQYLRWFEEWAEDFCRKKKKYVDIVKTYCREQDKSGNQ-RYCSRNGYDCTKTVRARGKYRMGNQCTKCLFACNPYVEWIENQRKQFDKQVKKY  cys5
XP_001349035.1  PQYLRWFEEWAEDFCRKRKKQLTDAIKNCREEDE-NGENRYCDLNGYDCKNTAKGKNKYKHDQECIKCSSVCIPFGPWIKNQKQEFEKQKNKY  cys4
XP_001349036.1  PQYLRWFEEWAEDFCRKKKKYVDIVKKFCRE--GENGKEKYCSLNGYDCTKTKLAVGKYRMGNQCTKCFFACYPYENWIEKQKEQFDKQKKIY  cys4
XP_001349219.1  PQYLRWFEEWAEDFCRKKNKKIKDVKTNCRDE-----KEKYCSGNGYDCRKTIYKKGKLVIGEHCTNCSVWCRLYEKWIDNQKKEFLKQKNKY  cys4
XP_001349434.1  PQYLRWFEEWAEDFCRKRKHKLQNAKEQCRGKNG-EDK--YCDLNGYDCKRTISAEKKLFPDSDCNKCSYSCIPFRTWIDNQKLEFLKQKNKY  cys4
XP_001349437.1  PQYLRWFEEWGEDFCRKKKKKVENAKKKCRGENN-DK---YCSRNGCDCEKTVRAKGKLRYGNRCTDCLYACYPYVHWIDKKKEEFDKQVKKY  cys4
XP_001349438.1  PQYLRWFEEWAEDFCRKKKHKLNDAIQKCRGEDK-YGKDRYCDLNGYDCEKTKRGRNMYRWDHKCTGCFLSCSHFRTWIDNQKLEFLKQKEKY  cys4
XP_001349514.1  PQYLRWFEEWAEDFCRKRKYKLENAIEKCRG-KT-KGE-KYCDLNGFDCTQTASGEKKFVKGHNCHNCSVTCIPFGPWIDNQKKEFLKQRNKY  cys4
XP_001349515.1  PQYLRWFEEWAEDFCRKKKKKIPNVKTNCRQVQR-G-KEKYCDRDGYNCDGTIRKQYIYRLDTDCTKCSLACKTFAEWIDNQKEQFDKQKQKY  cys4
XP_001349737.2  PQYLRWFEEWAEDFCRKKKKKLEKLEQQCRDYK----QNLYCSGNGYDCTKTIYKKGKLVIGEHCTNCSVWCRLYESWIDNQKLEFLKQKQKY  cys4
XP_001350409.1  PQYLRWFEEWAEDFCRKKKKKLKDVKRNCRDE-----TVKYCSGNGYDCTKTIYKKGKLVIGSECTKCSVWCRLYEKWIDNQKKEFLKQRNKY  cys4
XP_001350936.1  PQYLRWFEEWAEDFCRLRKHKLKDAKNKCRGDS---GNDRYCDLNRYDCTQTIRGNEHFVEKDDCKGCQYSCAHFVNWIDNQKLEFEKQKEKY  cys4
XP_001351079.1  PQFLRWFEEWAEDFCRLRKHKLKDAIDKCRTPK---GKEKYCDLNRYDCEQTIRGDHDFVEDDVCKGCQYSCSHFVNWIDNQKLEFDKQKRKY  cys3
XP_001351318.1  PQYLRWFEEWTEEFCRKKKKKLEDVIKKCRYDEN--NERKYCSRNGFDCKDTIRAQEKLVKGYDCHKCSVACDDFEPWIKNQKQEFEKQKGKY  cys4
XP_001351319.1  PQYLRWFEEWAEDFCRLRKHKLKDAIKKCRGKN---GEEKYCDLNRYDCKNTASGKHVFFEDFDCKDCQYSCAPFVDWIDNQKLEFLKQRKKY  cys4
XP_001351435.1  PQYLRWFEEWAEDFCRKKKKYVNIVKTYCRKK-DNSSEERYCSRNGFDCEKTKRAIGKLRYGKGCTDCFFACYPYEKWIDNKKKEFLKQKEKY  cys4
XP_001351437.1  PQYLRWFEEWAEDFCRKKKIYVGIVKTYCREKYKSGNEPRYCSRNGYDCTKTKRAIGKYRMGNQCISCLYACNPYVDWINNQKEQFDKQKQKY  cys4
XP_001351514.1  PQFLRWFEEWAEDFCRKKKKKLEKLEQQCRGVY--EGKERYCSRNGFDCEKTVNARGKVRMGKGCTDCFFACNPYIDWIEKQKEQFDKQKQKY  cys5
XP_001351515.1  PQYLRWFEEWAEDFCRKRKHKLKDVKTNCR-GEN--GTDRYCSGNGLDCTKTIRAKYIYAIGSECTKCSFLCGFYKKWIDNQKKEFLKQKKKC  cys4
XP_001351517.1  PQFLRWFEEWAEDFCRKKKKYVDIVKTYCREKDKY-GNERYCSGNGYDCTKTIYKKGRIVVGYECTSCSVWCRMYEKWIDNQKKEFLKQRNKY  cys4
XP_001351563.1  PQYLRWFEEWAEDFCRKRKYKLENAKEQCREKDK-YYQERYCSGNGYDCTQTVRAQEEYSMENNCHKCFFACNPFVKWLGNQKLEFDKQKKKY  cys4
XP_001351564.1  PQYLRWFEEWAEDFCRIRKHKLKNAKEQCREKYK-SGTDRYCSRNGYDCTQTIRGRNILVSDSECTNCSVVCTPFVKWIENKKLEFEKQKGKY  cys4
XP_001351877.1  PQFLRWFEEWAEDFCRKKKHKLKDVKTNCR-ND---EEQRYCSGNGFDCTKTIYKKGRIVIGEHCTKCSVWCRMYESWIDNQKKEFLKQKRKY  cys4
XP_001352242.1  PQYLRWFEEWAEDFCRKKKKYVDIVKTYCRG-KDKNSEERYCSRNGFDCEKTKRAIGKLRYGKQCISCLYACNPYVEWIDNQRKQFLKQKQKY  cys4
XP_002808728.1  PQFLRWFEEWAEDFCRKKKKYVDIVKTYCR----NETKGKYCSLNGYDCTKTKPAIGRLRMGNQCTKCFFACYPYEKWIDKKKEEFLKQKQKY  cys4
XP_002808906.1  PQYLRWFEEWAEDFCRKKKKKVQNLEQQCRGQD-ISGNQRYCSRNGYDCEKTKPAIGRLRMGKGCTDCFFACNPYIDWINNQRKQFDKQKNKY  cys4
```

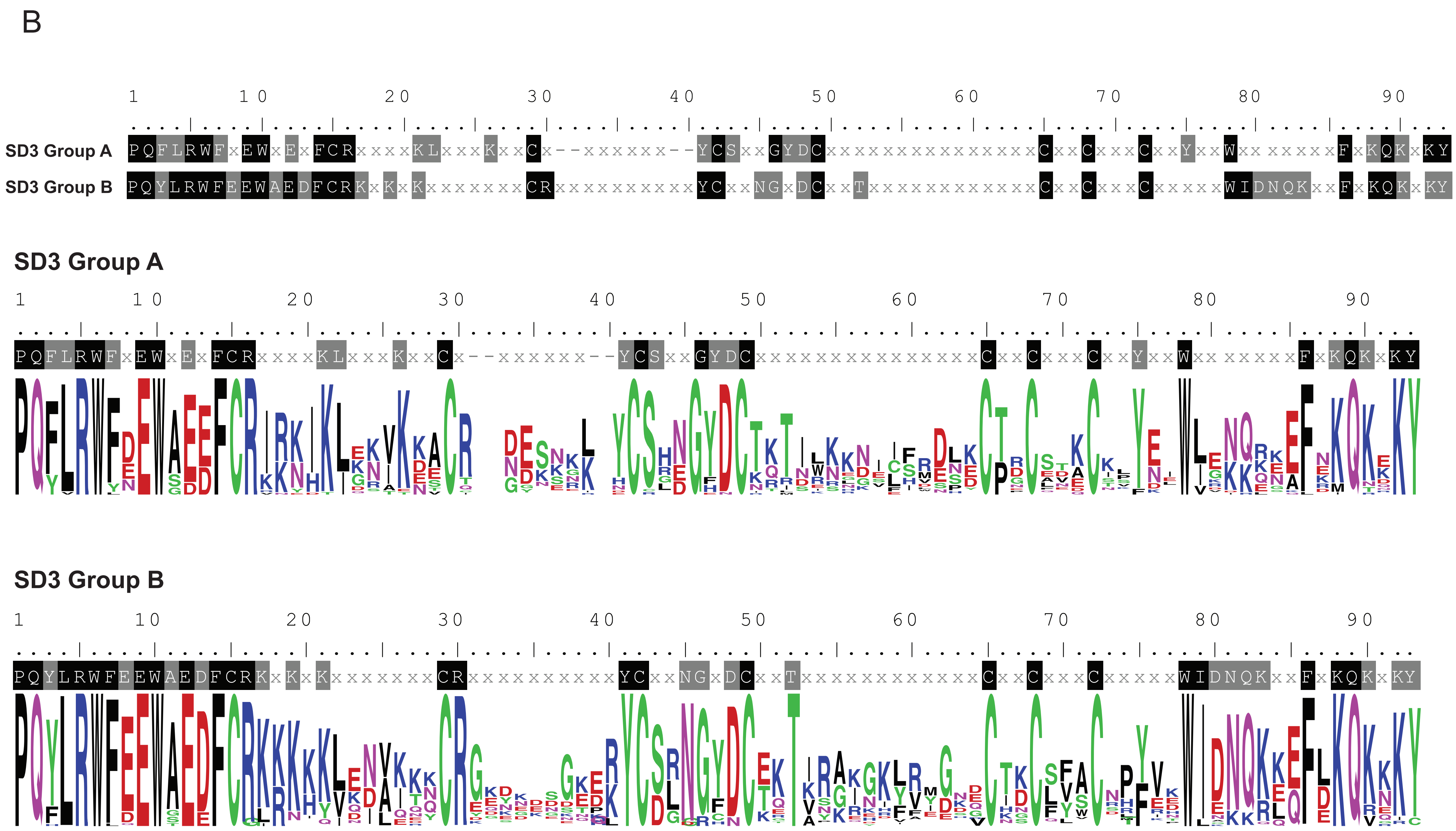
B
SD3 Group A
PQFLRWFxEWxExFCRxxxxKLxxxKxxCx--xxxxxx--YCSxxGYDCxxxxxxxxxxxxxxxCxxCxxxCxxYxxWxx xxxxxFxKQKxKY
SD3 Group B
PQYLRWFEEWAEDFCRKxKxKxxxxxxxCRxxxxxxxxxxYCxxNGxDCxxTxxxxxxxxxxxxCxxCxxxCxxxxxWIDNQKxxFxKQKxKY
SD3 Group A
SD3 Group B
