# Supplementary material for: Plasmodium falciparum Rosetting Epitopes Converge in the SD3-Loop of PfEMP1-DBL1α
Source: PLoS One. 2012 Dec 5;7(12):e50758. doi: 10.1371/journal.pone.0050758 (PMC3515580; doi:10.1371/journal.pone.0050758)
Supplement: Table S1 — PfEMP1 sequences on the peptide microarray. (DOC) [file pone.0050758.s007.doc]

**Table S1.**

**PfEMP1 sequences on the peptide microarray**

| Protein ID | Protein size on the array (aa) | Number of peptides (15aa) |
| --- | --- | --- |
| IT4var21 / FCR3S1.2 var1 NTS-DBL1α | 415 | 101 |
| IT4var60 / FCR3S1.2 var2 NTS-DBL1α-CIDR1α | 782 | 193 |
| IT4var9 / R29 NTS-DBL1α | 391 | 95 |
| TM284 NTS-DBL1α | 390 | 95 |
| VarO NTS-DBL1α | 398 | 97 |
| 3D7var4 NTS-DBL1α | 399 | 97 |

**NTS-DBL1α protein sequences**

# *IT4var21 / FCR3S1.2 var1 NTS-DBL1α*

MATSGGSGGTQDEDAKHVLDEFGQKVHDEVHGEAKNYVSELKGSLSLASILGETAFTVKSMQTESKYTELIEANSKRNPCKKDGKGNDVDRFSVKEQAGYDNKKMKCSNGDACAPFRRLHLCNKNFPNMNSYDSSKAKHDLLAEVCMAAKYEGESIKTHYPKYDSKYPGSDFPMCTMLARSFADIGDIIRGRDLYLGNKKKKQNGKETEREKLEQKLKEIFKKIHDNLKDKEAQKRYNGDEDPNFYKLREDWWTANRETVWGAMTCSKELDASSYFRATCSDTGQGPSQTHNKCRCDKDKGANAGKPKAGDGDVTIVPTYFDYVPQYLRWFEEWAEDFCRKKKKKLENLEKQCRGKDKSDEYRYCSRNGYDCEQTISRKGKVRMGKGCTDCFFACHSYENWIDNQRKQFDKQKKY

# *IT4var60 / FCR3S1.2 var2 (IT4var60) NTS-DBL1α-CIDR1α*

MAPKGRSTNEIELSARDVLENIGIGIYNQEKIKKNPYEQQLKGTLSNARFHDGLHKAADLGVIPGPSHFSQLYYKKHTNNTKYYKDDRHPCHGRQGKRFDEGQKFECGNDKIIGNSDKYGSCAPPRRRHICDQNLEFLDNNHTDTIHDVLGNVLVTAKYEGESIVNDHPDKKNNGNKSGICTSLARSFADIGDIVRGRDMFKPNDKDAVRHGLKVVFKKIYDKLSPKVQEHYKDVDGSGNYYKLREDWWTANRDQVWKAITYKAPQDANYFRNVSGTTMAFTSAGKCRHNDNSVPTNLDYVPQFLRWYDEWADDFCRIRNHKLQKVKDTCQGYNNSGYRIYCSGDGEDCTNILKQNFNIVSDFFCPSCKTECTNYKKWINKKQGEFNKQKKKYEKEINNIASNSDNTYDKKVYKTLKSMYPLDTKFVATLKEAPFCNNNNVDGIIDFNKPDDTFSSSTYCDSCPAFGVICENGTCTKVNEDTCSKMNVQVPKIITNKEDPTNIGILVSDDRVNVIPNELENGCKNTGIFKGIRKDEWSCKYLCNLDVCDLSHNKNNTHIDKRISIRVLFKRWLEYFLKDYSKLKKKLNSCTNNGKESICINECKKKCECVGKWAEEKRKEWEKVRKRFFNQYNVDDSLKSYEVKTFVNGNVDRSDIKNALNEGENLEALQDSDECIKPHNSKKDTCVKNDVVNILINRLKKKIDDCKIQHDNRTNQICCDELPESKEDNEDEEEEGEKKKNSKHLEETKEKKELDDNNFLDLCNNVKKYIEDNNKQISIQHK

*IT4var9 / R29 NTS-DBL1α*

MTPKRTSRTVNNLSATDVLEKIATGIYNQEKEKVYPYENELKGILSNAIFVDQLRKELNIESPGPSDSCSLDHKFHTNINTEYTEGRKPCYERNEKRFSNEGEAKCGSDKIRDYGIKSAGGACAPFRRQNLCDRNLEYLINKNTNTTHDLLGNVLVTAKYEGDSIVNNHPDKNSSGNKSSICTALARSFADIGDIVRGRDMFKPNDADKVEKGLQVVFGKIYNSLPSPAQKHYAHDDGSGNYYKLREDWWAINRKEVWKAITCRAPNEANFFRNISGNMKAFTSQGYCGHSETNVPTNLDYVPQFLRWFDEWAEEFCRIRKIKLENVKKECRDEPNNKYCSGDGHDCKRTYLKDNTIFIDLNCPRCENACSNYTKWIEIQRKQFDKQKRKY

*TM284 NTS-DBL1α*

MAPQKAAAPDYSSAKDAKELLDMIGEEVYKEKVKNAANDFREKLKGTLSQATFEEAPKEQQTPGNPCELKYQWHTNATRGKNYPCRTGTEKRFSEVSGGECDEKKIKDNKGKEGACAPYRRLHLCVRNLENISALDKINNDTLLADVCLAALHEGAAISADHGQYQQTNDSSQLCTMLARSFADIGDIIRGKDLYRGNNGKDKLEENLKKIFGNIYKDVTKGGKNVDALKTRYEDATGNYYKLREDWWNANRQEIWKALTCDAPNGDVHYFRKTCSMGQSHVNDKCRCLNGDPPTYFDYVPQYLRWFEEWAEDFCTKRKHKLQNAIKICRGDSGNDRYCDLNGYDCTKTARGKNKRFSNDECYKCSLPCDHFVPWIDNQQKEFEKQKKKY

PA*varO NTS-DBL1α*

MGSSHSTNDTKSPTLSESHKSARNVLENIGIKIYNQEIKKKNPYEQQLKGTLSRAQFVDALSSRYGYVRNSDGNSCNLDHLFHTNIKTGYNEGRKPCYGREQNRFDENAEAYCNSDKIRGNENNSNGTACAPPRRRHICDQNLEFLDNKNTNTTHDLLGNVLVTAKYEGNYIVNDHPDKNSNGNKSGICTSLARSFADIGDIVRGRDMFLPNKDDKVQKGLQVVFKKIYKSLTPEARKHYAHGDGSGNYSKLREDWWTINREQIWKALTCSAPYYADYFRKGSDGTLHFSSHGKCGHNEGAPPTYLDYVPQFLRWFEEWSEEFCRIKKIKIDKVKKECRDEQNKKYCSGDGHDCTQTNLSHNQIFVDLDCPRCQDQCIKYNEWIVKKLEEFYKQNLKY

*3D7var4 NTS-DBL1α*

MGNASSSEGEAKTPSLTESHNSARNILEGYAESIKEQASKDAKIHGHHLKGDLAKAVFRHPFSAYRPNYGNPCELDYRFHTNVWHRNAEDRNPCLFSRAKRFSNEGEAECNGGIITGNKGECGACAPYRRRHICDYNLHHINENNIRNTHDLLGNLLVMARSEGESIVKSHEYTGYGIYKSGICTSLARSFADIGDIIRGKDLYRRDSRTDKLEENLRKIFANIYKELKNGKKWAEAKEYYQDDGTGNYYKLREAWWALNRKDVWKALTCSAPRDAQYFIKSSVRDQTFSNDYCGHGEHEVLTNLDYVPQFLRWFEEWAEEFCRIKKIKLGKVKEACRDDSKKLYCSHNGYDCTKTIRNKDILSDNPKCTGCSVKCKVYELWLRNQRNEFEKQKKKYYK
