# Supplementary material for: Plasmodium falciparum Rosetting Epitopes Converge in the SD3-Loop of PfEMP1-DBL1α
Source: PLoS One. 2012 Dec 5;7(12):e50758. doi: 10.1371/journal.pone.0050758 (PMC3515580; doi:10.1371/journal.pone.0050758)
Supplement: Table S2 — PCR-primers and vectors used to generate NTSDBL1α-expression constructs. (DOC) [file pone.0050758.s008.doc]

**Table S2.**

PCR-primers and vectors used to generate NTSDBL1α-expression constructs

|  | Forward primer | Reverser primer | Vector | Restriction sites | Boundaries of the construct (aa) |
| --- | --- | --- | --- | --- | --- |
| NTS-DBL1α-IT4var60 | synthesized as recoded construct | synthesized as recoded construct | pQE70 | SphI, BglII | 1-482 |
| NTS-DBL1α-IT4var60 Subdomain 1 | *GC****ATG****C*GAGCACCT AAAGGCCGC | *AGATCT*GTATTT GTCGCTGTTGCC | pQE70 | SphI/BglII | 1-119 |
| NTS-DBL1α-IT4var60 Subdomain 2 | *GC****ATG***CGAGGTTCT TGCGCACCGCCG | *AGATCT*GCGGAA GTAGTTTGCATC | pQE70 | SphI/BglII | 120-272 |
| NTS-DBL1α-IT4var60 Subdomain 3 | *GC****ATG****C*ACGTCTCTGGCACTACCATG | *AGATCT*GTATTTCTTTTTCTGTTTGTTA | pQE70 | SphI/BglII | 273-393 |
| NTS-DBL1α-IT4var9 | *CC****ATG****G*GAACGCCAAAGCGTACAAGT | *GGATCC*ATATTTCCTTTTTTGCTTATC | pQE60 | NcoI, BamHI | 1-391 |
| NTS-DBL1α-PAvarO | *CC****ATG****G*GTTCCTCCCACTCCACC | *GGATCC*GTACTTCAGGTTCTGCTTGTA | pQE60 | NcoI, BamHI | 1-398 |
